# Role of Dicer-Dependent RNA Interference in Regulating Mycoparasitic Interactions

**DOI:** 10.1128/Spectrum.01099-21

**Published:** 2021-09-22

**Authors:** Edoardo Piombo, Ramesh R. Vetukuri, Anders Broberg, Pruthvi B. Kalyandurg, Sandeep Kushwaha, Dan Funck Jensen, Magnus Karlsson, Mukesh Dubey

**Affiliations:** a Department of Forest Mycology and Plant Pathology, Swedish University of Agricultural Sciences, Uppsala, Sweden; b Department of Plant Breeding, Horticum, Swedish University of Agricultural Sciences, Lomma, Sweden; c Department of Molecular Sciences, Swedish University of Agricultural Sciences, Uppsala, Sweden; d National Institute of Animal Biotechnology, Hyderabad, Telangana, India; Broad Institute

**Keywords:** antagonism, biocontrol, *Clonostachys rosea*, gene regulation, mycoparasitism, RNA interference, small RNA

## Abstract

Dicer-like proteins (DCLs) play a vital role in RNA interference (RNAi), by cleaving RNA filament into small RNAs. Although DCL-mediated RNAi can regulate interspecific communication between pathogenic/mutualistic organisms and their hosts, its role in mycoparasitic interactions is yet to be investigated. In this study, we deleted *dcl* genes in the mycoparasitic fungus *Clonostachys rosea* and characterize the functions of DCL-dependent RNAi in mycoparasitism. Deletion of *dcl2* resulted in a mutant with reduced secondary metabolite production, antagonism toward the plant-pathogenic fungus *Botrytis cinerea*, and reduced ability to control Fusarium foot rot disease on wheat, caused by Fusarium graminearum. Transcriptome sequencing of the *in vitro* interaction between the *C. rosea* Δ*dcl2* strain and *B. cinerea* or F. graminearum identified the downregulation of genes coding for transcription factors, membrane transporters, hydrolytic enzymes, and secondary metabolites biosynthesis enzymes putatively involved in antagonistic interactions, in comparison with the *C. rosea* wild-type interaction. A total of 61 putative novel microRNA-like RNAs (milRNAs) were identified in *C. rosea*, and 11 were downregulated in the Δ*dcl2* mutant. In addition to putative endogenous gene targets, these milRNAs were predicted to target *B*. *cinerea* and F. graminearum virulence factor genes, which showed an increased expression during interaction with the Δ*dcl2* mutant incapable of producing the targeting milRNAs. In summary, this study constitutes the first step in elucidating the role of RNAi in mycoparasitic interactions, with important implications for biological control of plant diseases, and poses the base for future studies focusing on the role of cross-species RNAi regulating mycoparasitic interactions.

**IMPORTANCE** Small RNAs mediated RNA interference (RNAi) known to regulate several biological processes. Dicer-like endoribonucleases (DCLs) play a vital role in the RNAi pathway by generating sRNAs. In this study, we investigated a role of DCL-mediated RNAi in interference interactions between mycoparasitic fungus *Clonostachys rosea* and the two fungal pathogens *Botrytis cinerea* and Fusarium graminearum (here called mycohosts). We found that the *dcl* mutants were not able to produce 11 sRNAs predicted to finetune the regulatory network of genes known to be involved in production of hydrolytic enzymes, antifungal compounds, and membrane transporters needed for antagonistic action of *C. rosea*. We also found *C*. *rosea* sRNAs putatively targeting known virulence factors in the mycohosts, indicating RNAi-mediated cross-species communication. Our study expanded the understanding of underlying mechanisms of cross-species communication during interference interactions and poses a base for future works studying the role of DCL-based cross-species RNAi in fungal interactions.

## INTRODUCTION

Small RNAs (sRNAs) are a group of noncoding RNAs. They play a central role in gene silencing at the transcriptional level through chromatin modification and at the posttranscriptional level through targeted destruction of mRNAs, also known as RNA interference (RNAi) (1–5). Dicer-like protein (DCL) plays central role in RNAi by cleaving the double-stranded RNA precursors and single-stranded RNA precursors with hairpin structures to generate sRNAs, often ranging in size from 18 to 40 nucleotides, called small-interfering RNAs (siRNAs) and microRNAs (miRNAs; microRNA-like RNAs [milRNAs] in fungi), respectively. In fungi, the most studied RNAi pathways are mediated by siRNAs and milRNAs and are dependent on DCLs for biogenesis and are thus called Dicer-dependent RNAi. Dicer-independent RNAi, such as that mediated by dicer-independent small interfering RNAs (disiRNAs), has also been identified in the filamentous fungus Neurospora crassa (6).

Small-RNA mediated RNAi is an evolutionarily conserved process of self-defense triggered by a wide variety of exogenous nucleic acids such as invading viruses, transgenes, transposons, and plasmids (7, 8). In fungi, a role of sRNA-mediated RNAi pathways in genome defense against the insertion of repetitive transgenes during vegetative growth (quelling) and the sexual phase of the life cycle (meiotic silencing of unpaired DNA [MSUD]) was first reported in N. crassa (9–11). Since then, RNAi pathways and their role in genome defense against retrotransposon activity have been demonstrated in several fungal species with diverse lifestyles (8, 12–20). However, in some fungal species, such as Saccharomyces cerevisiae and Ustilago maydis, genes related to the RNAi pathways are absent (21, 22). In addition to the role of genome defense against transgenes, the fungal RNAi machinery generates a variety of sRNAs that are involved in the regulation of numerous biological processes through targeted gene silencing (8, 23). For instance, sRNAs (mainly milRNAs) are found to be differentially expressed in fungi during different growth phases, developmental stages, and environmental conditions, including those involved in host-pathogen interactions (24–34). Furthermore, sRNAs can move bidirectionally between the species and modulate cellular functions of recipient cells by hijacking their RNAi machinery. Thus, they play an important role in interspecies communication between closely interacting symbiotic organisms, including parasitic and mutualistic interactions (35–40). However, the role of sRNAs in parasitic fungus-fungus interactions is yet to be investigated.

The filamentous fungus *Clonostachys rosea* is a ubiquitous soilborne ascomycete with a complex lifestyle as a necrotrophic mycoparasite and saprotroph (41). *C. rosea* efficiently overgrows and kills its mycohosts such as *Botrytis cinerea* and Fusarium graminearum (41–43). During mycoparasitic interactions or exposure to the secreted factors from mycohosts, *C. rosea* induces expression of genes associated with the production of secondary metabolites, hydrolytic enzymes, and other secreted proteins (43–50). Furthermore, *C*. *rosea* induces expression of genes coding for membrane transporters to efflux the endogenous toxic compounds and exogenous metabolites that may come from interacting organisms during the interspecific interactions (49, 51, 52). The role of secreted proteins/enzymes, secondary metabolites, and membrane transporters in antibiosis and mycoparasitism in *C. rosea* is proven (42–44, 50, 53, 54); however, the role of RNAi in regulating the cellular regulatory network during such interactions has not yet been investigated.

The present work aims to (i) characterize the RNAi machinery in *C*. *rosea*; (ii) identify milRNAs that are key regulators of genes associated with the antagonistic/mycoparasitic activity in *C*. *rosea*, as well as their potential endogenous and cross-species gene targets; and (iii) investigate common or species-specific responses in sRNA-mediated gene regulation in *C*. *rosea* against mycohosts. We used the two important plant-pathogenic fungi *B*. *cinerea* and F. graminearum as different mycohosts, since they are taxonomically different from each other and represent different disease types on different crops. We hypothesized that (i) sRNAs regulate mycoparasitic interactions in *C*. *rosea* at endogenous and cross-species level and that (ii) *C*. *rosea* responds with both common and mycohost-specific reactions toward *B*. *cinerea* and F. graminearum. To test these hypotheses, we generated gene deletion and complementation strains of genes coding for DCL proteins (DCL1 and DCL2) in *C*. *rosea* and used a holistic approach (sRNA, transcriptome, and secondary metabolome analysis) to investigate the sRNA-mediated regulatory network and its influence on mycoparasitic fungus-fungus interactions at endogenous and cross-species level.

## RESULTS

### Identification and sequence analysis of the predicted RNAi machinery in *C*. *rosea*.

Genes coding for different protein components involved in the RNAi pathway were identified through BLAST analysis of *C*. *rosea* strain IK726 genome version 1 (41) and version 2 (55) using N. crassa and *Trichoderma atroviride* argonout (AGO), DCL, and RNA dependent RNA polymerase (RDR) gene sequences as queries. Two AGO (AGO1, protein ID CRV2G00002735; AGO2, protein ID CRV2G00000975), two DCL (DCL1, protein ID CRV2G00009872; DCL2 protein ID CRV2G00008135), and three RDR (RDR1, protein ID CRV2G00001186; RDR2, protein ID CRV2G00002170; RDR3, protein ID CRV2G00007201) genes were identified in the *C*. *rosea* genome. Analysis of the translated amino acid sequences for the presence of conserved modules identified the domains known to be present in DCL (DEXDc, HELICc, Dicer dimer, and RNase III), AGO (ArgoN, DUF, PAZ, ArgoL2, and PIWI), and RDR proteins (see Fig. S2B in the supplemental material). The characteristics of *C*. *rosea* AGOs, DCLs, and RDRs are presented in Table S1C.

Phylogenetic analyses using DCL, AGO, and RDR amino acid sequences revealed that *C*. *rosea* putative DCLs were most closely related to their homologs in *Acremonium chrysogenum*, with around 57% sequence identity, and the same was true for *C. rosea* homologs of AGO1 and AGO2, but with an identity around 51%. The three putative RDR genes were similar to their homologs in *A. chrysogenum* as well, with identities of 37, 42, and 55%, respectively. In the phylogenetic analyses, the putative DCLs of *C. rosea* diverged in two clusters separating the DCL1 and DCL2 from the analyzed species (see Fig. S2C), and the same was evident for AGO1 and AGO2 (see Fig. S2D). The tree generated from the RDR sequences formed by three main clusters, each containing one of the *C. rosea* proteins (see Fig. S2E). Our data therefore suggest that *C*. *rosea* contain two DCL, two AGO, and three RDR genes, with clear orthologs in related species.

### Generation of gene deletion and complementation strains.

To investigate the biological roles of RNAi in *C*. *rosea*, genes encoding DCL proteins were selected for gene deletions as they act upstream in the RNAi pathways. Single *dcl1* and *dcl2* deletion strains (Δ*dcl1* and Δ*dcl2*) were generated, and they were successfully complemented with *dcl1* and *dcl2*, respectively, to generate Δ*dcl1*+ and Δ*dcl2*+ complementation strains. Results describing validation of gene deletion and complementation strains are presented in Fig. S1. Phenotypic analyses experiments were performed with *C*. *rosea* wild-type (WT), *dcl* deletion strains (Δ*dcl1* and Δ*dcl2*) and their respective Δ*dcl1*+ and Δ*dcl2*+ complemented strains.

### Deletion of *dcl* affects growth, conidiation, antagonism, and biocontrol.

**FIG 1:**
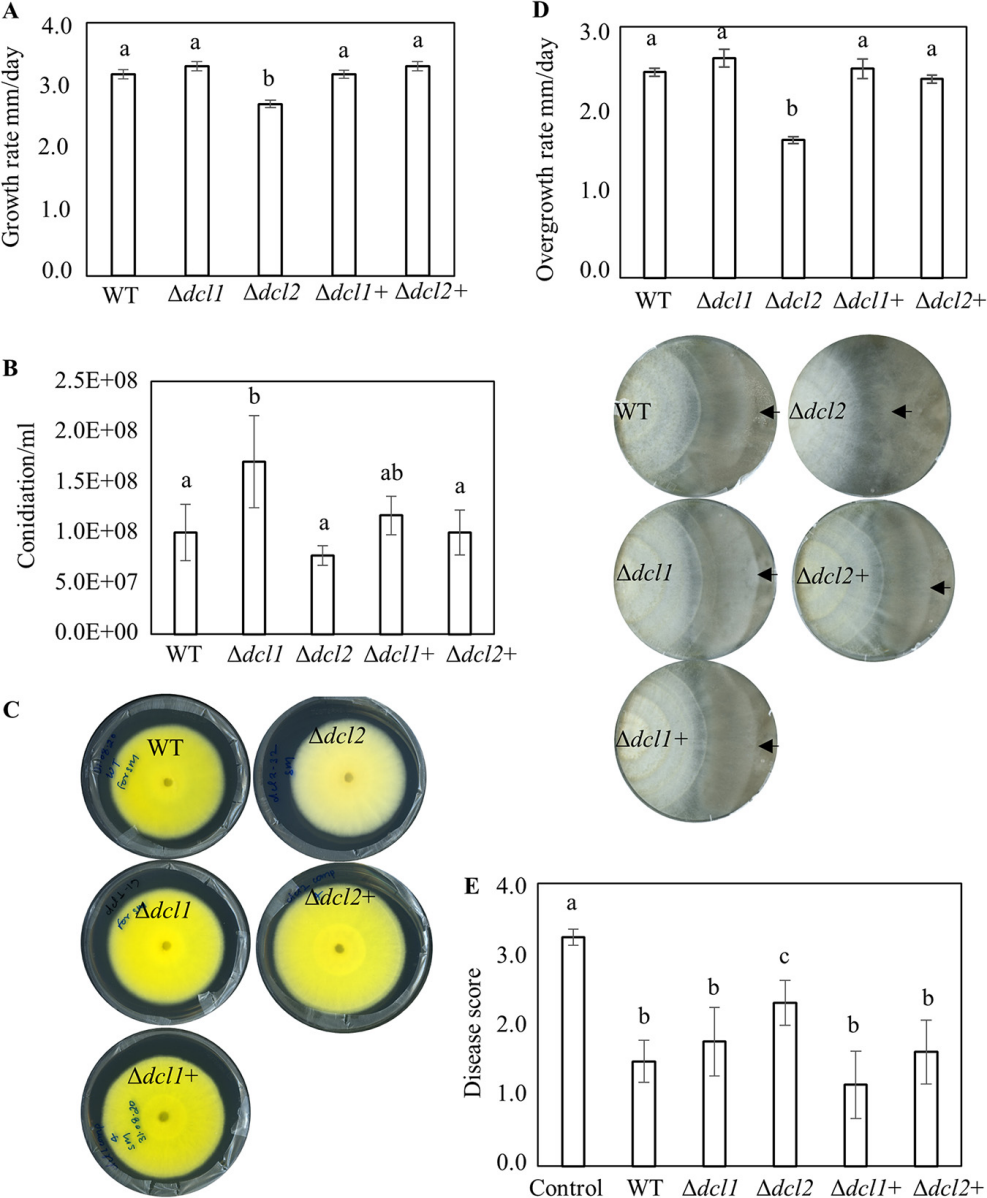
Phenotypic characterizations of *C*. *rosea* WT, deletion, and complementation strains. (A) Growth rate of WT, *dcl* deletion, and complemented strains. Strains were inoculated on PDA medium and incubated at 25°C, and the growth rate was recorded 5 days postinoculation (dpi). Error bars represent standard deviations based on four biological replicates. (B) Conidiation of WT, *dcl* deletion, and complementation strains on PDA medium 24 dpi. Conidia were harvested in equal volumes of water and were counted using a Bright-Line Haemocytometer according to the instructions of manufacturer. Error bars represent standard deviations based on four biological replicates. (C) Deletion of *dcl2* affects pigment production in *C*. *rosea*. Strains were inoculated on PDA medium and incubated at 25°C. The experiment was performed in four biological replicates, and photographs of representative plates were taken 16 dpi. (D) Dual culture assay to test antagonistic ability of *C*. *rosea* WT, deletion, and complementation strains against *B*. *cinerea*. Agar plugs of *C*. *rosea* strains (left side in the plate) and *B*. *cinerea* (right side in the plate) were inoculated on opposite sides in 9-cm-diameter agar plates, followed by incubation at 25°C. The growth rates (overgrowth) of *C*. *rosea* WT, deletion, and complementation strains on *B*. cinerea were measured from the point of mycelial contact. The experiment was performed in four replicates, and photographs of representative plates were taken 21 dpi of *C. rosea* strains. An arrowhead indicates the mycelial front of *C. rosea* strains. (E) *In vivo* assay to test the biocontrol ability of *C. rosea* strains against F. graminearum foot rot disease on wheat. Seeds were coated with *C. rosea* conidia and planted in moist sand together with a F. graminearum agar plug. Seedlings were harvested 21 dpi, and disease symptoms were scored on a scale from 0 to 4. The experiment was performed in five biological replicates with 15 plants in each replicate. Different letters indicate statistically significant differences based on Tukey HSD method at the 95% significance level.

The growth rate of the Δ*dcl2* strain was 14% lower (*P < *0.001) than the WT growth rate on potato dextrose agar (PDA), while no significant difference was found between the Δ*dcl1* strain and the WT (Fig. 1A). No significant difference in mycelial biomass (*P ≤ *0.36) between the *C*. *rosea* WT and the *dcl* deletion strains was found (see Fig. S3A). We quantified the conidiation of *C*. *rosea* WT and deletion strains 24 days postinoculation (dpi). At this time, the colony perimeter of each strain had reached the edge of the 9-cm petri dish. Conidium production for the Δ*dcl1* strain was 70% higher (*P = *0.014) than that of the WT, while no significant (*P = *0.75) difference in conidia yield was recorded in the Δ*dcl2* strain (Fig. 1B). Complementation Δ*dcl1*+ strains showed partial restoration of the conidial production phenotype observed in Δ*dcl1.* Morphological examination during growth on PDA revealed that the Δ*dcl2* strain had reduced ability to produce yellow pigment, while this phenotype remained unaffected in the Δ*dcl1* strain (Fig. 1C). No other marked difference in colony morphology was observed between the WT and the *dcl* deletion strains.

An *in vitro* dual culture assay was used to test whether deletion of *dcl1* or *dcl2* affected the antagonistic ability of *C. rosea*. No differences in growth rate of F. graminearum or *B*. *cinerea* were recorded during *in vitro* dual plate confrontation with either of the *dcl* deletion strains, compared to the WT (see Fig. S3A). However, a reduced ability (*P < *0.001) to overgrow *B*. *cinerea* was observed in Δ*dcl2* strains compared to the WT (Fig. 1D). The growth rate of Δ*dcl2* strains displayed 33% reduction on *B*. *cinerea* mycelium (overgrowth rate) compared to the growth rate of WT (Fig. 1D). In contrast, overgrowth of F. graminearum was not compromised in either of the deletion strains (see Fig. S3A). However, a change in F. graminearum color (pigment) was visible at the bottom side of the Δ*dcl2* mutant*-*F. graminearum interaction zone (see Fig. S3A). In contrast to *in vitro* antagonism tests, a bioassay for biocontrol of fusarium foot rot diseases on wheat caused by F. graminearum displayed a significant 56% increase (*P = *0.023) of disease severity in wheat seedlings previously seed coated with the Δ*dcl2* strain compared to seedlings from seeds coated with *C. rosea* WT (Fig. 1E). However, disease symptoms on seedlings from seeds coated with Δ*dcl1* strains showed no significant difference compared to the WT.

### Analysis of metabolites.

**FIG 2:**
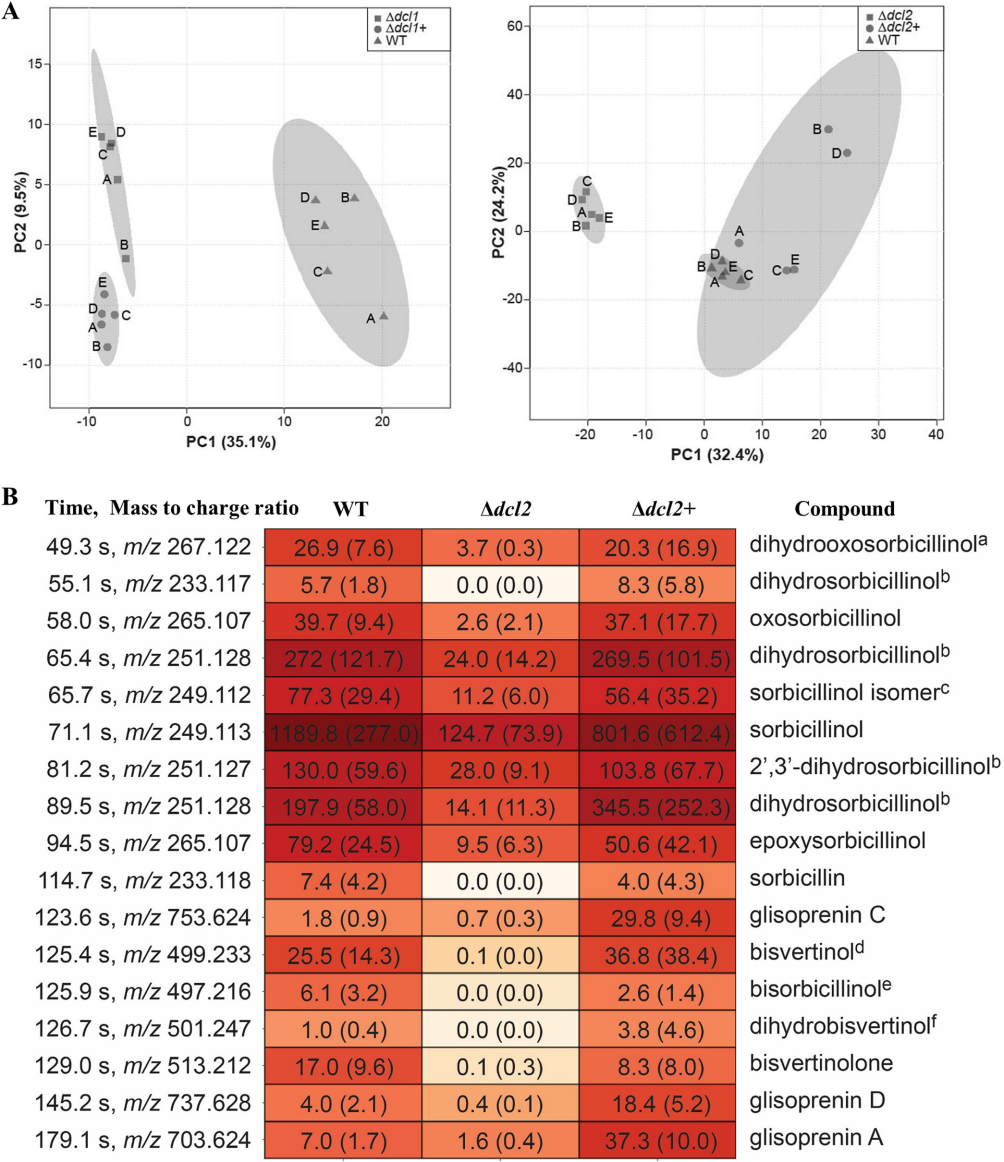
UHPLC-MS analysis of cultures of *C. rosea* WT and deletion strains. (A) PCA of UHPLC-MS data from analysis of metabolites produced by *C. rosea* WT and mutant (Δ*dcl1*, Δ*dcl2*, Δ*dcl1+*, and Δ*dcl2+*) strains. Shaded areas indicate 95% confidence regions. (B) Retention times, mass-to-charge ratios (*m/z*), extracted-ion chromatogram peak areas, and tentative identification by UHPLC-MS and UHPLC-MS/MS of 17 metabolites produced in significantly smaller amount in Δ*dcl2* mutants compared to the WT and restored in the compared Δ*dcl2+* strain (ANOVA FDR <0.01). The compound at 125.4 s was comparably underproduced and restored also in the *Δdcl1* strains. Ions are [M+H]^+^ except for the compound at 55.1 s, which is [M+H-H_2_O]^+^. The peak areas shown are average peak areas × 10^−3^ with standard deviations in brackets. The heatmap is based on sum-normalized and 10-logaritmized peak areas. Labels in panel A: A, may also be dihydroepoxysorbicillinol; B, proposed to be four different isomers of dihydrosorbicillinol; C, has the same *m/z* as sorbicillinol but different MS/MS data; D, may also be bisvertinoquinol or isobisvertinol; E, may also be bislongiquinolide or bisorbicillinolide or trichodimerol or trichotetronine; and F, may also be isodihydrobisvertinol.

The metabolites produced by the WT, *dcl* deletion, and complementation strains were analyzed by ultrahigh-performance liquid chromatography/mass spectrometry (UHPLC-MS) and UHPLC-tandem MS (UHPLC-MS/MS) (see Table S2). When analyzing the UHPLC-MS data by principal-component analysis (PCA), the samples from the Δ*dcl1*, Δ*dcl1+*, and WT strains grouped separated from each other (Fig. 2A, left) and, likewise, Δ*dcl2* and WT samples clustered separately (Fig. 2A, right). The Δ*dcl2+* samples, however, clustered with the WT samples, indicating restoration of metabolite production in Δ*dcl2+* strains. Two compounds were present in significantly smaller amounts in the Δ*dcl1* strain, and their production was restored in Δ*dcl1*+ strains, along with 15 further compounds (analysis of variance [ANOVA], false discovery rate [FDR] ≤ 0.01; see Fig. S3B and Table S2). Fifty-four metabolites were present in significantly smaller amounts in the Δ*dcl2* strain compared to the WT; at the same time, their production was restored in the Δ*dcl2+* strain (ANOVA, FDR ≤ 0.01; see Fig. S3B and Table S2). Seventeen of these compounds were tentatively identified or assigned to a compound class by UHPLC-MS, UHPLC-MS/MS, and database mining (Fig. 2B; see also Fig. S3C). Most of these substances were monomeric or dimeric hexaketides of the sorbicillin type (e.g., sorbicillin, sorbicillinol, oxosorbicillinol, epoxysorbicillinol, and bisvertinolone), whereas three glisoprenins (I, III, and IV) also were identified. The identification of some of these compounds is outlined below.

Sorbicillin was tentatively identified as a compound eluting at 114.7 s with [M+H]^+^ *m/z* 233.118, with two major fragment ions, *m/z* 95.049 and *m/z* 165.054, corresponding to bond cleavage on either side of the side chain carbonyl (see Fig. S3C). The ion at *m/z* 95.049 was diagnostic for all monomeric and dimeric sorbicillin-type compounds containing a hexa-2,4-diene-1-one motif. Fragment ions corresponding to the ion with *m/z* 165.054 discussed above were important for all monomeric sorbicillin type compounds, and related fragment ions were frequently found with additional loss of CO and/or water, depending on the respective compound structure. The compound eluting at 71.1 s, with [M+H]^+^ *m/z* 249.113, was tentatively identified as sorbicillinol based on such fragment ions (see Fig. S3C), and the two compounds at 58.0 s and 94.5 s, both with [M+H]^+^ *m/z* 265.207, were suggested to be oxosorbicillinol and epoxysorbicillinol, respectively, based on differences in fragment ions (see Fig. S3C). Five compounds in Fig. 2B gave *m/z* values which, after database mining, suggested that they were vertinolide or hydroxyvertinolide, hexaketides similar to the sorbicillins but with a lactone head-group instead of the aromatic ring or unsaturated cyclohexanone of sorbicillin-type compounds. In MS/MS, however, the vertinolide-type compounds did not yield fragment ions supporting their structures. Instead, MS/MS data suggested that these compounds were novel dihydrosorbicillinols or oxo/epoxy-dihydrosorbicillinol, respectively.

A large number of dimeric compounds of the sorbicillin-type are known (56), and several share the same molecular formula. These substances are dimerized by several different biosynthetic mechanisms, including Diels-Alder cycloaddition, Michael-type addition reactions, and formation of hemi-ketals. The compound eluting at 129.0 s, with [M+H]^+^ *m/z* 513.212 (in accordance with the compound bisvertinolone) gave two major fragment ions at *m/z* 249.111 and *m/z* 265.107, both [M+H]^+^, corresponding to the constituting monomeric compounds of bisvertinolone, i.e., sorbicillinol and oxosorbicillinol, respectively (see Fig. S3C). This pattern was observed for all putative dimeric sorbicillin-type compounds, i.e., in UHPLC-MS/MS analyses, these compounds fragmented to yield ions of the presumed constituting monomeric compounds, and related ions after loss of CO and/or water (see Fig. S3C). The formation of these fragment ions is possible for dimeric compounds formed by many different mechanisms, and therefore it was difficult to identify these by MS/MS without access to authentic reference compounds or very detailed information about the MS/MS behavior of these compounds. Therefore, several alternative identities are listed in Fig. 2B for some of the dimeric compounds. The polyhydroxy terpenes glisoprenin A, C, and D were identified based on the *m/z* of their respective [M+H]^+^ ions, supported by the *m/z* of fragment ions (loss of multiple water molecules) detected in UHPLC-MS/MS.

### Transcriptome analysis of *Clonostachys rosea* WT and *dcl* deletion strains.

To gain insights into the molecular mechanisms associated with the altered phenotypes of *C*. *rosea dcl* deletion strains, transcriptomes of *C*. *rosea* WT, Δ*dcl1*, and Δ*dcl2* were analyzed by RNA-seq during the interactions with *B*. *cinerea* and F. graminearum. An average of 20.5 million clean reads was obtained for each treatment. Since the sequences contained read pairs from both the interacting species, the reads originating from *C*. *rosea* or interacting mycohosts were identified by mapping to *C*. *rosea*, *B*. *cinerea*, or F. graminearum genomes. During the *C. rosea*-*B. cinerea* interaction, 24% of reads, on average, were mapped to *C. rosea* genes, while 58% of reads were assigned to *C. rosea* in the *C. rosea*-F. graminearum interaction. Summary data for transcriptome sequencing and mapping are presented in Table S3.

**FIG 3:**
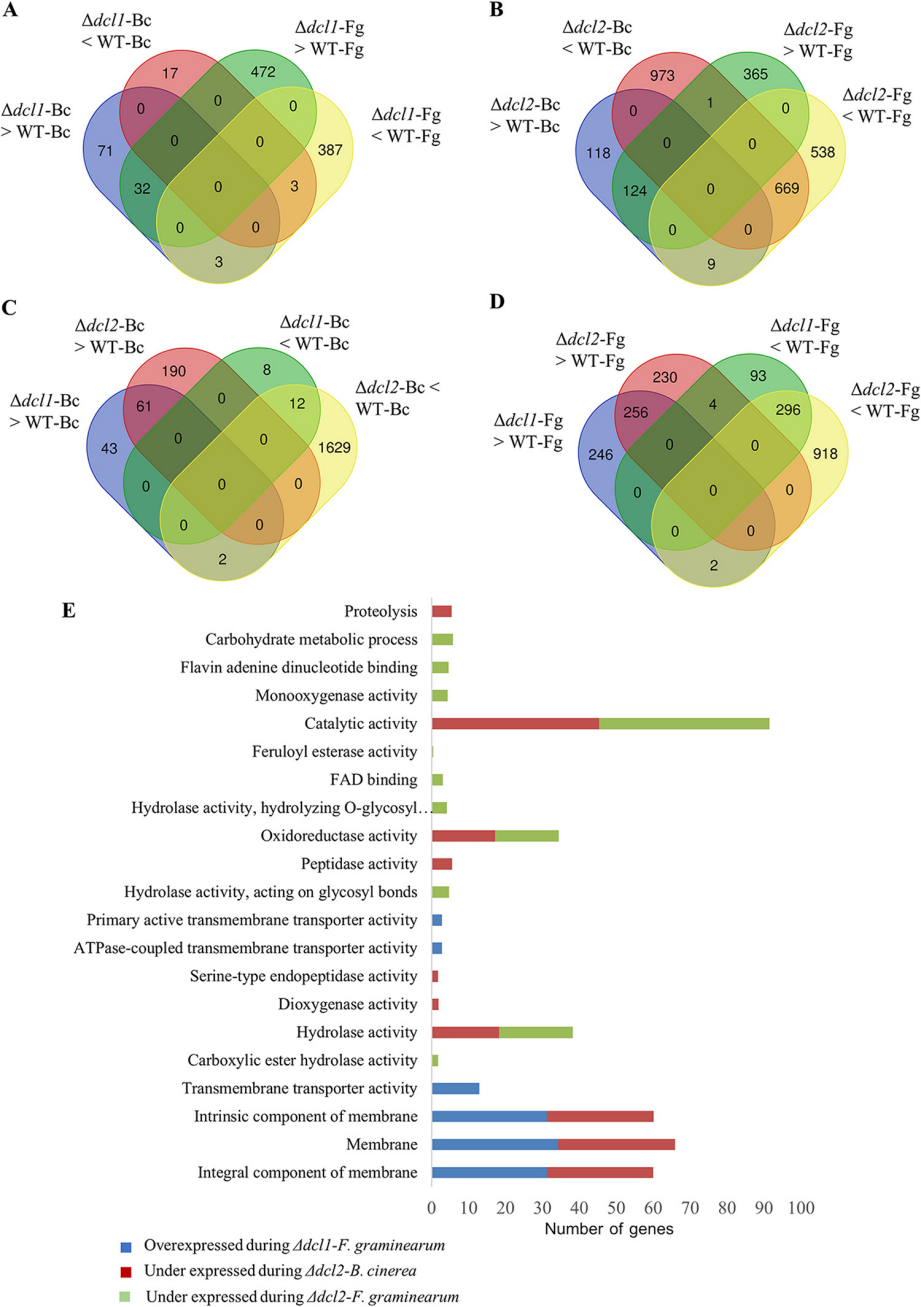
Transcriptome analysis of *C*. *rosea* WT and *dcl1* and *dcl2* deletion strains during the interactions with *B. cinerea* (Bc) and F. graminearum (Fg). (A) Venn diagram showing the common and species-specific DEGs in the Δ*dcl1* mutant against *B. cinerea* and F. graminearum. (B) Venn diagram showing the common and species-specific DEGs in the Δ*dcl2* mutant against *B. cinerea* and F. graminearum. (C) Overlap between DEGs in the Δ*dcl1* and Δ*dcl2* mutants against *B. cinerea*. (D) Overlap between DEGs in Δ*dcl1* and Δ*dcl2* mutants against F. graminearum. (E) Gene Ontology terms enriched in the differentially expressed *C. rosea* genes during the interactions.

Compared to the *C. rosea* WT, the analysis identified 126 differentially expressed genes (DEGs; 106 upregulated and 20 downregulated) in the Δ*dcl1* strain against *B*. *cinerea*, while this number was much higher against F. graminearum, where 897 genes (504 upregulated and 393 downregulated) were differentially expressed (see Table S4). Among these, a majority of genes were uniquely expressed in the respective interaction, since only 32 and 3 genes were commonly upregulated and downregulated, respectively, against both the mycohosts (Fig. 3A). The deletion of *dcl2* affected the expression pattern of a higher number of genes compared to the deletion of *dcl1*. In the Δ*dcl2* strain, in comparison to the WT, totals of 1,894 (251 upregulated and 1643 downregulated) and 1,706 (490 upregulated and 1216 downregulated) genes were differentially expressed against *B*. *cinerea* and F. graminearum, respectively (see Table S4). In contrast to the Δ*dcl1* strain, where a relatively lower proportion of genes (15.7% against *B*. *cinerea*; 43.7% against F. graminearum) were downregulated, a higher proportion (87% against *B*. *cinerea*,73% against F. graminearum) of DEGs in the Δ*dcl2* strain were downregulated. Among the upregulated genes in the Δ*dcl2* strain, 124 genes were commonly upregulated, while 118 genes and 365 genes, respectively, were uniquely upregulated against *B*. *cinerea* and F. graminearum. Among downregulated genes, 669 were common, while 973 and 538 genes, respectively, were unique against *B*. *cinerea* and F. graminearum (Fig. 3B).

The numbers of DEGs overlapping in Δ*dcl1* and Δ*dcl2* strains during the interactions with a common mycohost were determined (Fig. 3C and D). Among genes that were upregulated in Δ*dcl1* or Δ*dcl2* strains against *B*. *cinerea*, 61 were common, while 45 (41%) and 190 (76%) were uniquely upregulated in Δ*dcl1* and Δ*dcl2* strains, respectively. However, the number of genes downregulated in both mutants against *B. cinerea* was 12. During contact with F. graminearum, similar numbers of genes were upregulated in the two mutants (246 in the Δ*dcl1* strain, 230 in the Δ*dcl2* strain, and 256 in both strains), while the numbers of downregulated genes were greater in the Δ*dcl2* strain (93 in the Δ*dcl1* strain, 918 in the Δ*dcl2* strain, and 296 in both strains) (Fig. 3C and D).

GO enrichment analysis was performed to evaluate which processes were most affected in the *dcl* gene deletion mutants. Our results showed that a higher number of GO terms were significantly enriched in *C. rosea* genes under expressed in the Δ*dcl2* strain compared to the whole transcriptome. In the molecular function category, we found that terms such as catalytic activity (GO:0003824), hydrolase activity (GO:0016787), and oxidoreductase activity (GO:0016491) were commonly (against both the mycohosts) enriched (*P ≤ *0.05) among downregulated genes in the Δ*dcl2* strain, indicating a role of these genes in mycoparasitism-related functions in *C*. *rosea* (Fig. 3E). In contrast, other GO terms were only enriched against one of the two mycohosts. This was the case for the protein catabolism terms peptidase activity (GO:0008233) and proteolysis (GO:0006508), specifically enriched during the Δ*dcl2* mutant-*B*. *cinerea* interaction. Carbohydrate metabolism-related terms such as carbohydrate metabolism process (GO:0005975) and hydrolase activity acting on glycosyl bond (GO:0016798) were characteristic for the Δ*dcl2* mutant-F. graminearum interaction (Fig. 3E).

### DCLs regulate genes with a predicted function during fungus-fungus interactions in *Clonostachys rosea*.

Since the absence of DCL2 affected the production of secondary metabolites, antagonism, and biocontrol of *C*. *rosea*, we performed an in-depth analysis of genes with a reported function during interspecific interactions in *C. rosea*, including membrane transporters, enzymes involved in the biosynthesis of secondary metabolites, and hydrolytic enzymes. In addition, the expression pattern of genes coding for transcription factors and various components of the silencing machinery were analyzed. For each of these categories, there were more upregulated genes than downregulated ones in the Δ*dcl1* strain. An opposite pattern was evident in the Δ*dcl2* strain, where the number of upregulated genes in each category tended to be higher than that of downregulated ones, except for ABC transporters (Table 1; see also Table S5A).

**TABLE 1:** Number of differentially expressed genes in Δ*dcl1* and Δ*dcl2* mutants compared to wild-type *C. rosea* during the interaction with F. graminearum and *B*. *cinerea*

Type or function	No. of genes up- or downregulated							
	*C. rosea-* F. graminearum				*C. rosea-B. cinerea*			
	*Δdcl1* mutant		*Δdcl2* mutant		*Δdcl1* mutant		*Δdcl2* mutant	
	Up	Down	Up	Down	Up	Down	Up	Down
MFS transporters	26	16	12	64	5	1	6	99
ABC transporters	14	0	10	6	1	0	4	3
SM biosynthesis	45	38	27	99	7	1	13	127
Chitinases	0	0	3	3	1	1	1	3
Transcription factors	24	6	31	28	5	1	17	56
Gene silencing machinery	4	0	4	1	0	0	1	3

### (i) Membrane transporters.

Deletion of *dcl2* affected the expression of 161 major facilitator superfamily (MFS) transporters in *C*. *rosea*. Among these, 12 MFS transporters were upregulated, and 64 were downregulated during interaction with F. graminearum, whereas 6 were upregulated, and 99 were downregulated during interaction with *B*. cinerea (Table 1; see also Table S5A). Interestingly, 10 downregulated and 1 upregulated MFS transporters genes in the Δ*dcl2* strain showed high sequence similarity (≥48% identity) with MFS transporters previously characterized for their involvement in efflux of secondary metabolites (polyketides, quinones, and polyketide/nonribosomal peptide hybrids) that are important for fungal virulence (Table 2). These included *apdF* (aspyridones efflux protein in *Colletothricum siamense*), *opS2* (quinone transporter in Aspergillus *udagawae*), *atB* (terreic acid efflux protein in F. oxysporum), *FUB11* (fusaric acid efflux pump in *Lachnellula suecica*), *FUBT* (efflux pump involved in export of fusaric acid in *F. culmorum*), *rdc3* (radicicol efflux pump in F. oxysporum), and *aflT* (aflatoxin efflux pump in *Phialocephala subalpine*) (57–60). Furthermore, a homolog of *FUS6* (fusarin efflux pump FUS6 in *Colletothricum fructicola*) was upregulated. However, none of the corresponding gene clusters were present in the genome of *C. rosea*, suggesting that these MFS transporters constitute resistance proteins activated as a defense against harmful, hitherto-unknown, secondary metabolites. Moreover, 22 MFS transporter genes were previously reported to be induced in *C*. *rosea* during the interactions with *B*. *cinerea* and F. graminearum (49). Nine of these MFS transporter genes were significantly downregulated in the Δ*dcl2* strain during the interactions with *B*. *cinerea* or F. graminearum (Table 2). In summary the Δ*dcl2* mutant showed downregulation of transporters with predicted function in secondary metabolite export and putative detoxification.

**TABLE 2:** Differential expression patterns of selected genes in *C. rosea* Δ*dcl1* and Δ*dcl2* mutant strains during interactions with *B. cinerea* or F. graminearum compared to those of WT *C*. *rosea*

Gene ID	Log_2_FC expression^*a*^				Comment(s)
	Δ*dcl1* (Bc)	Δ*dcl1* (Fg)	Δ*dcl2* (Bc)	Δ*dcl2* (Fg)	
Differentially expressed MFS transporter genes identical to previously characterized MFS transporters					
CRV2G00017900	−0.36	**−1.94**	0.23	**−5.05**	*mfs212* (ID 50% with *apdF* [PKS-NRPS transport])
CRV2G00017824	0.36	−0.68	0.21	**−1.54**	*mfs* (ID 48% OpS2 [Quinone transport])
CRV2G00015530	−0.21	−1.89	0.09	**−2.28**	*mfs* (ID 59% with atB [terreic acid transport])
CRV2G00015418	0.02	**−1.61**	**−1.09**	**−1.56**	*mfs* (ID 60% with FUB11 [polyketide transport])
CRV2G00004817	0.53	**−1.6**	**−4.04**	**−2.92**	*mfs506* (ID 57% with FUBT [polyketide transport])
CRV2G00002357	−0.4	−1.26	**−1.69**	**−1.96**	*mfs533* (ID 70% with rdc3 [polyketide transport])
CRV2G00016200	0.12	−0.69	**−2.31**	**−2.18**	*mfs530* (ID 60% with rdc3 [polyketide transport])
CRV2G00004939	0.22	−1.76	**−2.09**	**−3.04**	*mfs534* (ID 80% with rdc3 [polyketide transport])
CRV2G00019617	1.94	4.06	**1.59**	**3**	*mfs595* (ID 77% with FUS6 [polyketide transport])
CRV2G00011170	0.95	0.17	0.14	**−3.32**	*mfs602* (ID 60% with aflT [polyketide transport])
CRV2G00005334	0.05	−5.44	**−4.55**	**−5.94**	*mfs589* (ID 70% with aflT [polyketide transport])
					
Reduced expression of MFS transporters that were induced in *C*. *rosea* against *B*. *cinerea* or F. graminearum					
CRV2G00004685	0.32	−0.79	0.62	**−1.57**	*mfs464*
CRV2G00005389	−0.81	−0.75	**−1.79**	**−1.38**	*mfs271*
CRV2G00018263	−0.37	−0.79	−0.74	**−2.14**	*mfs524*
CRV2G00011170	−0.03	−1.18	0.14	**−3.32**	*mfs602*
CRV2G00012180	1.12	**−2.65**	−1.45	**−2.9**	*mfs166*
CRV2G00015972	−0.06	**−2.26**	**−1.77**	**−2.3**	*mfs205*
CRV2G00004853	0.45	−1.45	**−2.37**	**−2.27**	*mfs104*
CRV2G00004939	0.22	**−1.76**	**−2.09**	**−3.04**	*mfs534*
CRV2G00018885	−0.39	−1.22	**−3.55**	**−2.63**	*mfs24*
					
Differentially expressed polyketide and nonribosomal peptide synthetase genes					
CRV2G00011222	−0.67	0.01	0.03	**−1.88**	*pks14*
CRV2G00013582	0	−1.43	−0.03	**−1.61**	*pks23*
CRV2G00015413	0.75	**−2.28**	**−1.86**	**−2.96**	*pks12*
CRV2G00015415	1.09	**−2.7**	**−3.22**	**−3.15**	*pks2*
CRV2G00018696	−0.92	−0.63	−0.13	**−4.97**	*pks6*
CRV2G00018222	0.03	−1.43	**−2.43**	**−1.79**	*pks22*
CRV2G00004952	0.11	**1.88**	0.74	**1.54**	*nrps*
CRV2G00005605	0.65	**2.73**	**1.95**	**2.33**	*nrps*
CRV2G00012656	0.18	**1.82**	**1.95**	**2.17**	*nrps16*
CRV2G00015275	−0.15	−0.7	0.76	**−2.06**	*nrps*
CRV2G00016915	0.67	**−1.91**	**−3.07**	**−3.17**	*nrps*
CRV2G00014896	0.25	1.44	1.26	**1.68**	*nrps9*
CRV2G00005211	0.26	**−1.62**	**−3.74**	**−2.3**	Indole
CRV2G00002084	**4.33**	0.12	**5.24**	−0.84	Terpene
					
Differentially expressed transcription factor genes identical to previously characterized transcription factors					
CRV2G00004759	−0.69	−0.32	**−1.75**	−1.02	ID 60% with FGR27
CRV2G00006707	−0.01	−0.9	**−1.62**	−1.31	ID 73% with CCAAT-binding subunit HAP3
CRV2G00015419	0.29	−0.95	**−2.22**	**−1.73**	ID 53% with sorbicillin regulator YPR2
CRV2G00011734	0.32	**1.81**	0.56	1.41	ID 79% with *abaA*
CRV2G00011385	0.19	−0.46	**2.58**	1.16	ID 57% with CTF1
CRV2G00016352	0.73	**1.51**	0.47	1.3	ID 65–70% SUC1
CRV2G00019080	**1.98**	**2.1**	1.16	**1.5**	ID 65% with SUC1
CRV2G00019116	0.9	**2.32**	1.01	**2.2**	ID 70% SUC1
CRV2G00016935	−0.74	−0.22	**−1.69**	−0.7	ID 69% with *prtT*
CRV2G00018531	−0.21	−0.48	**−2.12**	−1.35	ID 61% with sterol uptake control 2
CRV2G00019093	−0.38	0.43	**−1.5**	−0.14	ID 60% with GAL4
					
Differentially expressed chitinases and *N*-acetylhexosaminidase genes					
CRV2G00001280	−0.08	−0.85	**−3**	**−1.67**	Chitinase *ech42*
CRV2G00003425	−0.3	**−1.54**	**−3.6**	**−3.2**	Chitinase *ech37*
CRV2G00018858	−0.01	−0.06	**−1.9**	**−1.82**	Chitinase *chia5*
CRV2G00017631	−0.07	0.16	0.62	**2.51**	Chitinase
CRV2G00006887	0.82	**2.18**	0.92	**1.75**	Chitinase *ech58*
CRV2G00011101	−0.3	−0.13	**2.25**	**2.1**	Chitinase *chic1*
CRV2G00002927	−0.21	−0.42	**−1.76**	−0.78	NAG
CRV2G00012950	−0.14	−0.43	**−2.5**	**−2.26**	NAG
					
Differentially expressed genes associated with gene silencing machinery					
CRV2G00000975	0.2	0.1	1.2	**1.9**	Argonaute2-like
CRV2G00016556	0.2	**2.1**	0.4	1.3	Chromatin remodeling protein
CRV2G00012165	0.2	**4**	−0.4	**4.3**	Histone deacetylase
CRV2G00007951	0.4	0.4	1	**1.6**	Histone deacetylase
CRV2G00006603	0.9	**2.3**	**2.4**	**2.3**	RNA helicase
CRV2G00007159	0.6	**1.6**	0.5	1	RNA helicase
CRV2G00001612	−0.6	0.1	**−1.6**	**−1.8**	RNA helicase
CRV2G00012613	−0.7	0.9	**−2.4**	0.1	RNA helicase
CRV2G00009762	0	0.9	**−1.7**	−0.6	RNA-directed RNA polymerase

a Significant differences are indicated in boldface letters. FDR < 0.05 in combination with a log_2_ fold change (log_2_FC) of >1.5 or <−1.5 was considered to define differentially expressed genes. Bc, *B. cinerea*; Fc, F. graminearum.

In contrast to the expression pattern of MFS transporters, a higher number of ATP-binding cassette (ABC) transporter genes was upregulated in both the deletion strains, but specifically against F. graminearum, where 14 and 10 genes, respectively, were upregulated in the Δ*dcl1* or Δ*dcl2* mutant (Table 1). Of 19 ABC transporters that were differentially regulated in Δ*dcl2*, 5 upregulated and 1 downregulated belonged to the multidrug resistance protein (MDR) subfamily, 3 downregulated and 1 upregulated belonged to the multidrug resistance-associated protein (MRP) subfamily, and 4 upregulated and 1 downregulated belonged to pleiotropic drug resistance protein (PDR) subfamily (see Table S5A).

### (ii) Secondary metabolite biosynthetic genes.

Genes associated with secondary metabolite production are often arranged in biosynthetic gene clusters (BGCs) that consist of genes coding for core enzymes typically nonribosomal peptide synthetase (NRPS), polyketide synthase (PKS), or terpene cyclase, together with genes coding for additional proteins, including modifying enzymes, transporters, and transcription factors (61). We used antiSMASH to predict the biosynthetic gene clusters in *C*. *rosea* and identified 33 NRPS BGCs, 29 PKS BGCs, 7 BGCs for terpenes and 7 BCGs for NRPS-PKS hybrids, and 1 BGC for indole and betalactone biosynthesis.

**FIG 4:**
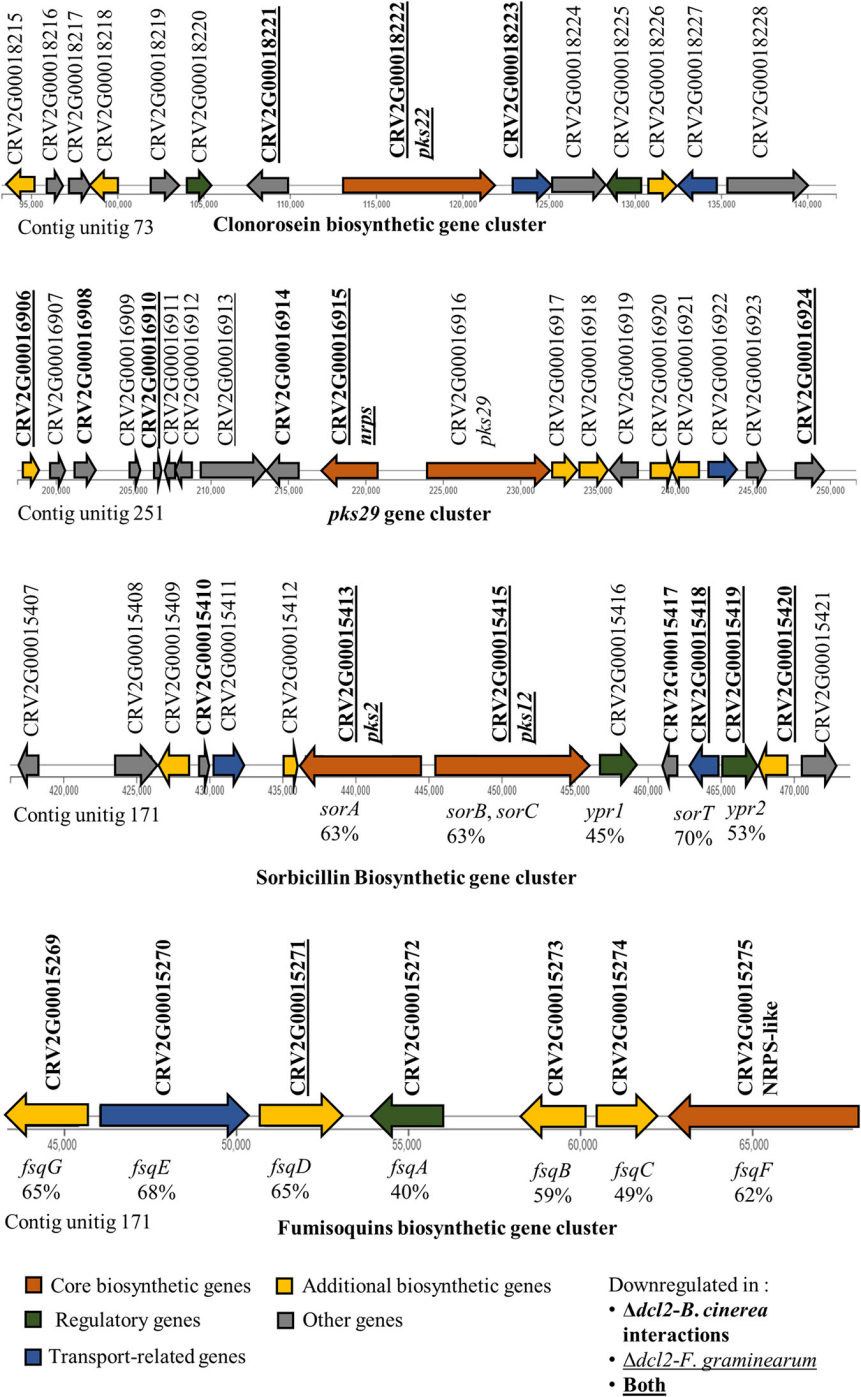
Expression of predicted *C. rosea* gene clusters of clonorosein, *pks29*, sorbicillin, and fumisoquins. Gene IDs in boldface letters indicate downregulated genes during *Δdcl2* mutant-*B. cinerea* interactions. Underlining indicates downregulated genes during *Δdcl2* mutant-F. graminearum interactions. Boldfacing and underlining indicates genes that were downregulated against both mycohosts. The gene names for the sorbicillin and fumisoquin gene clusters were assigned by comparison to Trichoderma reesei and Aspergillus fumigatus, respectively (63, 73). A minimum query coverage of 80% was required in the comparison, and the maximum E value was fixed at 1 × 10^7^.

Gene expression analysis of both Δ*dcl1* and Δ*dcl2* mutants identified a total of 230 DEGs predicted to be part of BGCs involved in secondary metabolite biosynthesis. Among the BGCs, the core biosynthetic genes in eight NRPS, five PKS, one terpene, and one indole BGCs were differentially regulated in the Δ*dcl2* mutant against *B*. *cinerea* or *F*. *graminearum* (Table 2; see also Table S5A). Interestingly, NRPS and PKS BGC core genes showed expression patterns opposite to each other since NRPS BGC core genes were mostly upregulated in the Δ*dcl2* mutant, whereas PKS BGC core genes were downregulated (Table 2). Among the downregulated core genes of PKS BGCs were the three PKS genes *pks22*, *pks2*, and *pks12*, reported to be part of previously identified BGCs responsible for the production of clonorosein and sorbicillin in *C*. *rosea* and *T*. *reesei*, respectively (Fig. 4) (50, 62). Sorbicillin is the precursor for sorbicillinol, which is in turn necessary for other sorbicillinoid compounds (63), explaining the low production of these substances by the Δ*dcl2* mutant.

### (iii) Transcription factors.

The transcriptome analysis further identified 128 differentially expressed genes predicted to encode transcription factors in the Δ*dcl1* and Δ*dcl2* strains (Table 1; see also Table S5A). We identified 11 transcription factors genes that were differentially expressed in the Δ*dcl1* strain and/or in the Δ*dcl2* strain and showed >50% sequence identity with genes previously characterized for their role as transcriptional regulators. CRV2G00011734 was upregulated in the Δ*dcl1* strain and showed identity with the conidiophore development regulator gene *abaA* (64, 65), whereas CRV2G00016352, CRV2G00019080, and CRV2G00019116, also upregulated, showed identity with the sucrose metabolic gene *suc1*, shown to be associated with mitotic and meiotic cell division in fission yeast (66). The genes CRV2G00004759, CRV2G00006707, and CRV2G00015419, downregulated in the Δ*dcl2* mutant, showed identity with transcription factor genes *fgr27*, *hap3*, and *ypr2*, shown to be involved in regulating growth and secondary metabolite production (62, 67, 68) (Table 2). In summary, the Dicer-dependent control of transcription factor gene expression was to a large degree mycohost specific, with no transcription factors differentially expressed against both mycohosts in the Δ*dcl2* mutant. Moreover, among the identified transcription factors, there were many homologs of genes known to have a role in regulating secondary metabolism and growth.

### (iv) Glycosyl hydrolase families 18 and 20.

The *C*. *rosea* genome contains 13 genes coding for enzymes with predicted chitinase (glycoside hydrolase family 18 [GH18]) activity (44), 6 of which were differentially regulated in the Δ*dcl2* mutant against *B*. *cinerea* or F. graminearum (see Table S5A). Among these, CRV2G00001280 (*ech42*), CRV2G00003425 (*ech37*), and CRV2G00018858 (*chiA5*) were downregulated against both the mycohosts, while CRV2G00017631, CRV2G00006887 (*ech58*), and CRV2G00011101 (*chiC1*) were upregulated against both the mycohosts (Table 2). Furthermore, the *C*. *rosea* genome contains two genes (CRV2G00002927 and CRV2G00012950) coding for predicted *N*-acetylhexosaminidases (NAG; GH20), the expression of which was downregulated in the Δ*dcl2* strain against *B. cinerea* (both genes) and F. graminearum (only CRV2G00012950). In summary, many glycoside hydrolases with a known role in degrading mycohost cell walls were downregulated in the Δ*dcl2* mutant after contact with the mycohosts.

### (v) Genes associated with gene silencing machinery.

To investigate an effect of *dcl1* and *dcl2* deletions on various protein components involved in the gene silencing machinery through chromatin modification in *C*. *rosea*, Blast2GO was used to identify genes encoding RNA helicases, chromatin remodeling proteins, histone deacetylases, and histone methyltransferases. We identified 118 genes (excluding DCL, AGO, and RDR), including 67, 23, 18, and 3 genes coding for RNA helicases, chromatin remodeling proteins, histone deacetylases, and histone methyltransferases, respectively (see Table S5B). Deletion of *dcl1* did not cause differential expression in the Δ*dcl1* mutant-*B*. *cinerea* interaction, whereas during contact with F. graminearum we detected upregulation of two RNA helicase genes (CRV2G00006603 and CRV2G00007159), one gene coding for a chromatin remodeling protein (CRV2G00016556) and a histone deacetylase gene (CRV2G00012172), while one histone deacetylase gene (CRV2G00012172) was downregulated (Table 2). During the Δ*dcl2*-*B*. *cinerea* interaction, one RNA helicase gene (CRV2G00006603) was upregulated, and two RNA helicases (CRV2G00001612 and CRV2G00012613), as well as an RNA-directed RNA polymerase (CRV2G00009762) were downregulated. Conversely, during the Δ*dcl2* mutant*-*F. graminearum interaction, two histone deacetylases (CRV2G00012165 and CRV2G00007951), one RNA helicase gene (CRV2G00006603), and one gene coding for an Argonaute protein (CRV2G00000975) were upregulated, whereas one RNA helicase (CRV2G00001612) gene was downregulated (Table 2). In summary, many genes involved in chromatin modification and gene silencing are affected by the deletion of the *dcl* enzymes, particularly *dcl2.* Most of these, including an Argonaute protein, are upregulated, possibly due to the diminished presence of regulating sRNAs in the mutants.

### Analysis of sRNAs characteristics in the *Clonostachys rosea* WT and the *dcl* deletion strains.

To investigate the effect of sRNAs on transcriptional regulation in *C*. *rosea*, sRNA libraries from *C*. *rosea* WT, Δ*dcl1*, and Δ*dcl2* strains interacting with *B*. *cinerea* or F. graminearum were sequenced. The sequencing produced 16 million reads per sample on average. Between 61 and 72% of these read pairs were composed of nonstructural RNAs, including rRNA, tRNA, snoRNA, and snRNA, and were excluded from the further analysis. The remaining subset of reads that were 18 to 32 nucleotides (nt) long were used for alignment to the genomes of *C. rosea*, *B. cinerea*, and F. graminearum. A summary of sRNA characteristics and their alignment to the respective genome is presented in Table S6A in the supplemental material. sRNAs mapping exclusively to the *C*. *rosea*, *B. cinerea*, or F. graminearum genome (unique sRNAs) were selected for further analysis. On average 42% of sRNA reads from *C*. *rosea*-*B. cinerea* interactions were aligned uniquely to one of the two organisms, while this percentage was only 18% for *C. rosea*-F. graminearum interactions. This is plausible because *C. rosea* is evolutionarily closer to F. graminearum (both belong to order *Hypocreales*) than to *B. cinerea*.

**FIG 5:**
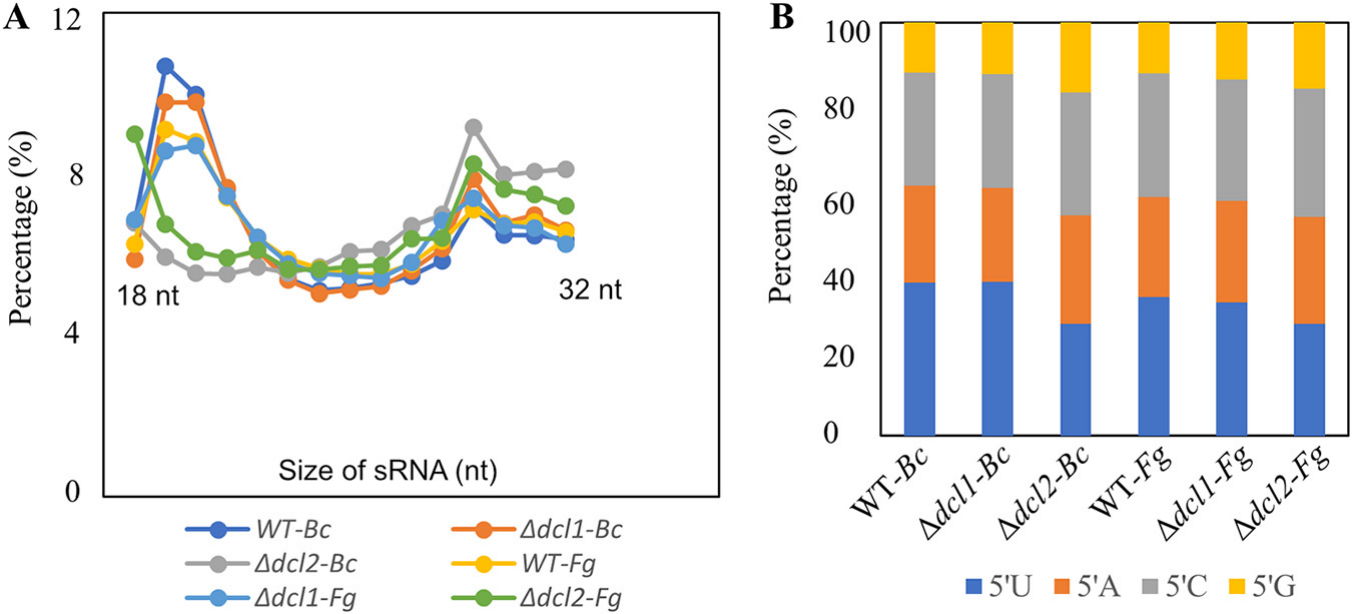
sRNA characteristics in *C. rosea* wild-type (WT) and *dcl* deletion strains. (A and B) Length distribution (A) and 5′ end nucleotide preference (B) of nonstructural sRNAs produced by *C. rosea* WT and *dcl* deletion strains during the interactions with F. graminearum (Fg) and *B. cinerea* (Bc). Only sRNAs between 18 and 32 nt in length are considered.

We compared the characteristics of sRNAs produced in the Δ*dcl1* and Δ*dcl2* mutants to those of the WT. The analysis of length distribution showed a significant reduction in sRNAs with a size of 19 to 22 nt in the Δ*dcl2* compared to the WT, while no difference in sRNA abundance was found between the Δ*dcl1* and WT strains (Fig. 5A). The analysis of the 5′ terminal nucleotide composition showed a reduced proportion of reads (27%) with 5′ end uracil (5′-U) in the Δ*dcl2* strain, compared to a 32 to 37% proportion of reads with 5′-U from the WT and Δ*dcl1* strains (Fig. 5B). The origin of sRNAs was not significantly affected by the deletion of *dcl* genes, with most reads mapping to coding sequences (CDSs; 49%), followed by intergenic regions (25%), promoters (12.3%), 3′ untranslated region s (UTRs) (8%), introns (4%), and 5′ UTRs (1.5%). A higher proportion (83.5%) of sRNAs was mapped with the sense orientation, rather than the antisense one, similar to what was reported in previous studies in F. graminearum and T. atroviride (20, 69), and this might be due to by-products of mRNA degradation. However, the relative proportion of sRNAs mapping to the antisense direction was reduced from an average of 17.5% during WT-*B*. *cinerea* interaction to 14.3% during Δ*dcl2* strain-*B*. *cinerea* interaction (see Table S6A).

### (i) milRNA prediction in *Clonostachys rosea*.

Mirdeep2 analysis predicted 61 milRNAs in *C*. *rosea* with lengths between 18 and 25 nt, and they were named cro-mir’s. These milRNAs originated from a variety of positions in the genome including promoters, introns, CDSs, and UTRs, but mainly (28 of 61) from intergenic regions (see Table S6B). The expression of 15 cro-mir’s was common against both mycohosts, whereas 29 and 17 cro-mir’s were expressed specifically during interaction with *B. cinerea* or F. graminearum, respectively (see Table S6B). Interestingly, no cro-mir was found to be differentially expressed in the Δ*dcl1* mutant during the interspecific interactions, while 11 cro-mir’s were significantly downregulated in the Δ*dcl2* mutant during interaction with both mycohosts (Table 3). This downregulation was confirmed through stem-loop RT-qPCR (Table 3). A single milRNA (cro-mir-23) was identified as upregulated in the Δ*dcl2* mutant in the RNA-seq analysis but downregulated according to stem-loop RT-qPCR.

**TABLE 3:** Differentially expressed cro-mir’s, their lengths, and their loci of origin^*a*^

milRNA identifier	Sequence (5′–3′)	Length (nt)	Log_2_FC				Origin
			RNA-seq		Stem-loop RT-qPCR		
			Δ*dcl2* (Bc)	Δ*dcl2* (Fg)	Δ*dcl2* (Bc)	Δ*dcl2* (Fg)	
cro-mir-1	TAGAATTCGGGGTAGAAT	18	−7.90	−7.15	−8.22	−9.43	Intergenic
cro-mir-2	TAGAATTCGGGGTAGAATG	19	−8.70	−8.23	−3.33	−10.94	Intergenic
cro-mir-3	TTAGCCTCGAGACTTTGCA	19	−8.28	−7.23	−5.85	−2.16	3′ UTR
cro-mir-4	TCAGCCTCGAGACTTTGCC	19	−8.47	−6.25	−2.18	−2.92	3′ UTR
cro-mir-5	TTGCAATGATTTGCATTTCGC	21	−3.52	−2.61	−3.54	−1.31	Intergenic
cro-mir-6	TAGGACTCGAGTAGTTATAAC	21	−4.39	−4.70	−2.05	−1.75	Intergenic
cro-mir-9	TCGGACGTATATTGACTACTC	21	−3.88	−3.22	−2.87	−2.71	Promoter
cro-mir-10	TCGGTGGGATGTTTGAGACT	20	−3.80	−2.59	−3.43	−3.21	Promoter
cro-mir-11	TAGAGTTTTTGGAGATGCT	19	−5.22	−4.68	−5.31	−3.05	Promoter
cro-mir-13	TTCTTCCTTGATGCGTCCC	19	−7.92	−7.74	−5.64	−6.07	3′ UTR
cro-mir-23	CTGGCAGGTATGGTCGTAGATG	22	+2.68	+2.18	−2.09	−3.10	Intergenic
cro-mir-36	TCAAACACAATTAGCGGTC	19	−7.30	−6.21	−4.26	−3.50	Intergenic

a nt, nucleotides; UTR, untranslated region; Bc, *B. cinerea*; Fc, F. graminearum.

### (ii) Identification of cro-milRNAs endogenous gene targets.

Twenty-one putative endogenous gene targets were identified for the 11 cro-mir’s downregulated in Δ*dcl2* (Table 4). Eight gene targets were commonly upregulated in Δ*dcl2* during the interaction with *B*. *cinerea* and F. graminearum, while seven and six gene targets were uniquely upregulated during the interactions with *B. cinerea* and F. graminearum, respectively (Table 4). Among the predicted gene targets, several had putative regulatory roles: CRV2G00015277, CRV2G00002266, and CRV2G00002043 were predicted to encode putative transcription factors, CRV2G00001868 encodes an ATP-dependent helicase, while CRV2G00004332 and CRV2G00008014 encode a GTP binding protein and a GTPase with a putative role in signal transduction. Moreover, CRV2G00014914 was located in a secondary metabolite gene cluster and might have a role in regulating secondary metabolism (Table 4).

**TABLE 4:** Endogenous putative gene targets in *C. rosea*, their expression patterns, and their predicted functions

milRNA identifier	Gene target	Expression log_2_FC^*a*^		Target gene family	Characterized/putative function
		Δ*dcl2* (Bc)	Δ*dcl2* (Fg)		
cro-mir-3	CRV2G00002264	**1.08**	**1.42**	Serine/threonine-protein kinase (Gin4)	Septin ring assembly, intracellular signal transduction
cro-mir-5	CRV2G00013335	**1.39**	**1.25**	Unknown	Unknown function
cro-mir-5	CRV2G00015277	**2.54**	**3.52**	Transcription factor	60S ribosome biogenesis
cro-mir-10	CRV2G00015277	**2.54**	**3.52**	Transcription factor	60S ribosome biogenesis
cro-mir-11	CRV2G00015277	**2.54**	**3.52**	Transcription factor	60S ribosome biogenesis
cro-mir-13	CRV2G00001868	**1.95**	**2.72**	Helicase	Chromatin remodeling
	CRV2G00002266	**1.81**	**1.98**	Transcriptional regulator *prz1*	Regulates the expression of the Pmc1 ATPase Ca^2+^ pump
cro-mir-36	CRV2G00013380	**2.42**	**3.36**	ATPase	ATPase activity
	CRV2G00005499	**1.38**	**1.8**	Unknown	Unknown function
	CRV2G00000111	**1.95**	**2.69**	Unknown	Unknown function
	CRV2G00014914	**1.21**	0.82	Oxidation-reduction process	Part of secondary metabolite BGC
cro-mir-1	CRV2G00003756	**1.06**	0.89	tRNA ligase	Protein biosynthesis
cro-mir-2	CRV2G00003756	**1.06**	0.89	tRNA ligase	Protein biosynthesis
cro-mir-3	CRV2G00008014	**1.12**	0.23	GTPase-activating protein 2	Signal transduction
cro-mir-6	CRV2G00002043	**1.12**	0.99	Transcription factor	Regulation
cro-mir-3	CRV2G00009307	**1.26**	0.81	Sterol *O*-acyltransferase 2	Cholesterol metabolic process
cro-mir-11	CRV2G00009307	**1.26**	0.81	Sterol *O*-acyltransferase 2	Cholesterol metabolic process
cro-mir-3	CRV2G00011242	**1.26**	0.75	Oxidoreductase	Oxidation-reduction
cro-mir-4	CRV2G00011242	**1.26**	0.75	Oxidoreductase	Oxidation-reduction
cro-mir-13	CRV2G00004332	**1.06**	0.43	GTP-binding protein	Ribosome biogenesis
cro-mir-1	CRV2G00005300	0.69	**1.38**	Unknown	Unknown function
cro-mir-4	CRV2G00004339	0.48	**1.03**	SNF2 RNA helicase	Chromatin remodeling
cro-mir-9	CRV2G00004339	0.48	**1.03**	SNF2 RNA helicase	Chromatin remodeling
cro-mir-10	CRV2G00004339	0.48	**1.03**	SNF2 RNA helicase	Chromatin remodeling
cro-mir-11	CRV2G00000903	0.82	**1.03**	Unknown	Unknown function
cro-mir-36	CRV2G00000903	0.82	**1.03**	Unknown	Unknown function
cro-mir-10	CRV2G00011823	0.93	**1.21**	Choline-sulfatase	Hydrolase activity
cro-mir-36	CRV2G00011823	0.93	**1.21**	Choline-sulfatase	Hydrolase activity
cro-mir-4	CRV2G00012062	−0.18	**1.09**	Unknown	Unknown function
cro-mir-13	CRV2G00012781	0.3	**1.01**	Unknown	Unknown function

a Upregulated (FDR < 0.05 in combination with log_2_FC >1) gene targets are highlighted in boldface. Bc, *B. cinerea*; Fc, F. graminearum.

### (iii) Cross-species gene target identification.

Using the criteria described for the endogenous gene target prediction, we identified 513 putative cross-species gene targets in *B*. *cinerea* (see Table S6C). Among these, the seven genes *bcpls1*, *bcpka1*, *bcnoxA*, *bcste11*, *bccap9*, *bccrh1*, and *bcchsIV* were previously characterized for their role in growth and development, proteolysis, and consequently virulence (Table 5). Moreover, a gene encoding a *B. cinerea* homolog of SSAMS2 (BCIN_08g03180) was also among the putative targets, and this gene encodes a GATA transcription factor required for appressoria formation and chromosome segregation in Sclerotinia sclerotiorum (70). In addition, *bcnog1* and *bchts1* encoding proteins putatively involved in ribosome biogenesis, and *bcphy2* and *bchhk1* encoding signal transduction proteins were also identified as putative targets. Finally, three genes coding for a protein with a putative role in chitin recognition (*bcgo1*), chromatin remodeling (*bcyta7*), and intracellular trafficking and secretion (*bcvac8*) were also identified (Table 5).

**TABLE 5:** Most important cross-species putative gene targets in *B*. *cinerea* and F. graminearum, their expression pattern and putative function

milRNA identifier	Gene target transcript ID	Locus ID (gene name)	Expression (log_2_FC)	Target gene family	Characterized or putative function
*Botrytis cinerea*					
cro-mir-1, cro-mir-2, and cro-mir-6	XM_024690817	Bcin_01g09230 (*bcphy2*)	3.53	Protein kinase	Signal transduction
cro-mir-9	XM_024690817	Bcin_05g05430	3.38	Phospholipid methyltransferase	Lipid metabolic process (membrane lipid biogenesis)
cro-mir-13	XM_001553702	Bcin_02g04090	2.9	Fungal 1,3(4)-β-d-glucanases	Glucan catabolic process
cro-mir-13 and cro-mir-2	XM_001547426	Bcin_01g00360 (*bcerg1*)	2.74	Squalene monooxygenase	Sterol biosynthetic process
cro-mir-4	XM_001557947	Bcin_12g00180 (*bccap9*)	2.69	Aspartic proteases of fungal origin	Proteolysis, induced during infection
cro-mir-5	XM_001557734	Bcin_04g06150	2.29	Cyclase (Lanc-like super family)	Biosynthesis of lantibiotics
cro-mir-1 and cro-mir-2	XM_024693876	Bcin_07g01580 (*bcnog1*)	2.27	GTP-binding protein	Ribosomal large subunit biogenesis
cro-mir-4	XM_024693364	Bcin_06g01930 (*bcgo1*)	1.87	Chitin binding	Chitin recognition
cro-mir-5	XM_001561274	Bcin_01g06010 (*bccrh1*)	1.83	Glycosylphosphatidylinositol-glucanosyltransferase	Fungal cell wall biosynthesis
cro-mir-5	XM_024691832	Bcin_03g02630 (*bcste11*)	1.81	Protein kinase	Signal transduction, virulence
cro-mir-36	XM_024691483	Bcin_02g06930	1.67	1,3-β-d-Glucan synthase	Glucan biosynthesis
cro-mir-36	XM_001558808	BCIN_02g02410	1.61	Glycosyl hydrolase	Fungal-type cell wall polysaccharide metabolic process
cor-mir-11	XM_001551241	BCIN_14g02820	1.57	β-Glucan synthesis-associated protein	Fungal cell wall biosynthesis
cro-mir-11	XM_001550300	BCIN_05g00350 (*bcnoxA*)	1.57	NADPH oxidase (NOX)	Pathogenicity, fusion of conidial anastomosis tubes, and formation of sclerotia and conidia
cro-mir-4	XM_024690414	BCIN_01g03790 (*bcchsIV*)	1.54	Chitin synthase	Cell wall biosynthesis, development and pathogenicity
cro-mir-4	XM_024692792	BCIN_05g00540 (*bchhk1*)	1.47	Protein kinase	Signal transduction b
cro-mir-13 and cro-mir-2	XM_001551683	BCIN_09g06130 (*bcpls1*)	1.4	Tetraspanins	Appressorium development, host penetration
cro-mir-1 and cro-mir-2	XM_001547152	BCIN_12g05700	1.38	Cyclases	Biosynthesis of lantibiotics
cro-mir-36	XM_001554608	BCIN_08g03180	1.26	Transcription factor	Appressorium formation
cro-mir-36	XM_024694081	BCIN_07g04590 (*bchts1*)	1.2	Histidine-tRNA ligase	Translation, ribosomal structure, and biogenesis
cro-mir-4 and cro-mir-36	XM_024695521	BCIN_10g02810 (*bcyta7*)	1.13	Bromodomain-containing protein	Chromatin remodeling
ro-mir-1 and cro-mir-2	XM_024694912	BCIN_09g01210 (*bcchs1*)	1.11	Chitin synthase	Cell wall biosynthesis, virulence
cro-mir-13	XM_024697868	BCIN_16g01130 (*bcpka1*)	1.03	Serine/threonine kinases	Conidial germination, growth, and virulence
cro-mir-5	XM_024694566	BCIN_08g03270 (*bcvac8*)	1.02	Fungus-type vacuole membrane	Intracellular trafficking and secretion
					
Fusarium graminearum					
cro-mir-3	XM_011328464	FGSG_07067	1.41	Transcription factor	Virulence
cro-mir-4	XM_011319656	FGSG_02083	1.02	Transcription factor	Mycotoxin biosynthesis
cro-mir-5	XM_011317736	FGSG_00376	1.07	Ubiquinone oxidoreductase	Virulence
cro-mir-5	XM_011321023	FGSG_13747	1.03	Membrane transporter	Transmembrane transporter activity
cro-mir-5	XM_011329154	FGSG_07665	1.14	Membrane transporter	Transmembrane transporter activity
cro-mir-1 and cro-mir-2	XM_011319110	FGSG_11973	1.44	Membrane transporter	Transmembrane transporter activity
cro-mir-9	XM_011329717	FGSG_09686	1.58	Vesicle-mediated transport	Intracellular trafficking and secretion
cro-mir-6	XM_011326744	FGSG_06384	1.11	Vesicle-mediated transport	Intracellular trafficking and secretion

Thirty-five cross-species gene targets were predicted in F. graminearum as well. We identified three previously characterized virulence factors (FGSG_07067, FGSG_02083, and FGSG_00376) as putative targets of cro-mir-3, cro-mir-4, and cro-mir-5, respectively (Table 5). In addition, three membrane transporter genes (FGSG_13747, FGSG_13747, and FGSG_13747) and two genes coding for proteins with a putative role in intracellular trafficking and secretion (FGSG_09686 and FGSG_09686) were identified as putative targets (Table 5). In summary, several mycohost genes with a role in virulence, intracellular trafficking, secretion, and regulation were identified as putative targets of *C. rosea dcl2*-dependent milRNAs.

### *Botrytis cinerea* and *Fusarium graminearum* responded differently toward *Clonostachys rosea* WT and *dcl* deletion strains.

Transcriptome analysis of *B*. *cinerea* and F. graminearum was performed to investigate whether the deletion of *dcl* genes affects their response mechanism to *C*. *rosea*. Read pairs unique to *B*. *cinerea* from the *C*. *rosea*-*B. cinerea* interaction and unique to F. graminearum from the *C. rosea*-F. graminearum interaction were used in the analysis. From the total number of read pairs that originated from the *C. rosea*-*B. cinerea* or *C*. *rosea*-F. graminearum interactions, 25 and 23% reads were uniquely assigned to *B. cinerea* and F. graminearum, respectively (see Table S3).

**FIG 6:**
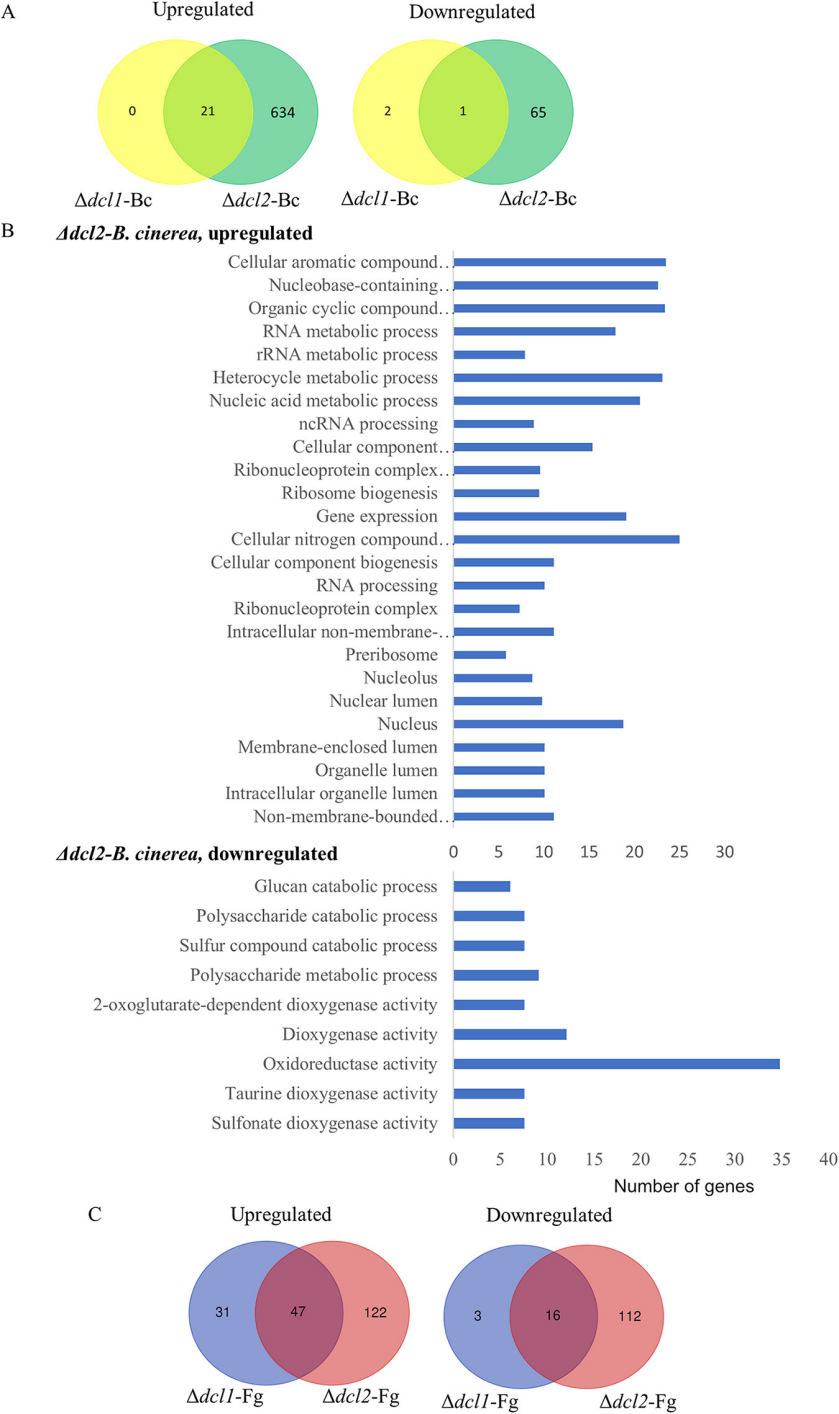
Transcriptome analysis of *B*. *cinerea* (Bc) and F. graminearum (Fg) during the interaction with *dcl1* and *dcl2* deletion strains compared to those of the WT. (A) Venn diagrams showing the overlap between upregulated and downregulated genes in the Δ*dcl1* and Δ*dcl2* strains during the interactions with *B. cinerea* compared to the WT. (B) Gene Ontology terms enriched in upregulated and downregulated genes in *dcl2* deletion strains during the interactions with *B. cinerea*. (C) Venn diagrams showing the overlap between up- and downregulated genes in Δ*dcl1* and Δ*dcl2* strains during interactions with F. graminearum compared to the WT.

In comparison to the WT-*B. cinerea* interaction, 24 genes (21 upregulated and 3 downregulated) were differentially expressed in *B. cinerea* during the Δ*dcl1* mutant-*B. cinerea* interaction. However, 721 genes were found to be differentially regulated (655 upregulated and 66 downregulated) in the interaction with the Δ*dcl2* mutant (Fig. 6A; see also Table S6C). The 21 *B*. *cinerea* genes that were upregulated against the Δ*dcl1* strain were also upregulated against the Δ*dcl2* strain (Fig. 6A). We specifically investigated genes coding for hydrolytic enzymes, transcription factors, membrane transporters, known virulence factors, RNA silencing component proteins, and genes that are part of secondary metabolite BGCs. During Δ*dcl1* mutant-*B*. *cinerea* interaction, one gene (BCIN_14g03930) coding for a known virulence factor and two genes coding for MFS transporters were upregulated, while two genes that were part of secondary metabolite BGCs were downregulated in *B*. *cinerea*. Deletion of *dcl2* induces increased expression of 12 genes previously characterized for their role in growth and development, virulence, and pathogenesis in *B. cinerea*. Among the other genes, we detected the upregulation of GTPases, kinases, chitinases, squalene monooxygenases, and genes involved in chitin synthesis and chitin recognition (Table 6).

**TABLE 6:** Differential expression patterns of selected genes in *B*. cinerea and F. graminearum during interaction with *Δdcl1* and *Δdcl2* mutants compared to those of wild-type *C. rosea* and the same mycohost

GenBank accession no.	Locus tag (gene ID)	Gene function	Expression (log_2_FC)^*a*^		Biological function
			*Δdcl1*	*Δdcl2*	
*Botrytis cinerea*					
XM_001547559	BCIN_02g08360 (bcfrq1)	Circadian oscillator	1.05	**2.03**	Virulence
XM_001550300	BCIN_05g00350 (bcnoxa)	NADPH oxidase complex	−0.39	**1.57**	Virulence
XM_001552181	BCIN_12g03770 (bcnop53)	Pre-rRNA processing factor	0.19	**1.59**	Fungal development and pathogenesis
XM_001555445	BCIN_03g06840 (bcnoxr)	Regulatory subunit of NOX (NADPH oxidase regulator)	−0.01	**1.56**	Differentiation and pathogenicity
XM_024691832	BCIN_03g02630 (bcste11)	MAPK triple kinase	0.16	**1.81**	Hyphal growth
XM_024693262	BCIN_06g00026 (mfsG)	Major facilitator superfamily transporter	−0.84	**−5.95**	Tolerance to glucosinolate-breakdown products, required for pathogenicity
XM_024697209	BCIN_14g03930 (bcltf1)	GATA transcription factor	**1.66**	**3.86**	Tolerance to oxidative stress, virulence
XM_024697551	BCIN_15g03390 (bcvel1)	Regulatory protein of the VELVET complex	0.13	**1.59**	Formation of oxalic acid, virulence
XM_024694938	BCIN_09g01620 (bccry2)	DNA photolyase	1.74	**3.57**	Negative regulation of filamentous growth and conidiation
XM_001561274	BCIN_01g06010 (bccrh)	Transglycosylase	0.00	**1.83**	Cell wall biogenesis, virulence
XM_024693846	BCIN_07g01300 (bcchsvii	Chitin synthase	0.06	**1.83**	Cell wall biogenesis, virulence
XM_024696504	BCIN_12g05360 (bcchsvi)	Chitin synthase	0.04	**1.66**	Cell wall biogenesis, Virulence
XM_001545464	BCIN_12g05370 (bcchsv)	Chitin synthase	−0.12	**1.63**	Cell wall biosynthesis
XM_024690414	BCIN_01g03790 (bcchsiv)	Chitin synthase	−0.15	**1.54**	Cell wall biosynthesis
XM_001554790	BCIN_03g09000	Septin GTPase	**2.87**	**5.60**	Cytoskeleton-dependent cytokinesis (septin ring)
XM_024693922	BCIN_07g02420	MFS transporters	−0.83	**2.99**	Xenobiotic transport
XM_024695797	BCIN_11g00800	Protein kinase CK2	1.43	**2.96**	Regulates various cellular processes
XM_024690261	BCIN_01g01760	Chitinase activity	0.06	**2.67**	Cell wall biosynthesis
XM_024696411	BCIN_12g03920	Chitin binding	0.35	**2.22**	Chitin recognition
XM_001549884	BCIN_01g02970	Chitin binding	0.00	**1.96**	Chitin recognition
XM_024693364	BCIN_06g01930 (bcgo1)	Chitin binding	−0.07	**1.87**	Chitin recognition
XM_001547426	BCIN_01g00360 (bcerg1)	Squalene monooxygenase	**1.55**	**2.74**	Sterol biosynthetic process
					
Fusarium graminearum					
XM_011317671	FGSG_00324 (*fgmyt3*)	Transcription factor	+1.05	**+1.52**	Fungal development and pathogenicity
XM_011318135	FGSG_00729 (*gzhmg005*)	Transcription factor	+0.99	**+1.56**	Virulence
XM_011320684	FGSG_10057 (*fgerb1*)	Transcription factor	+1.44	**+1.52**	Growth and pathogenicity
XM_011321826	FGSG_08617 (*gzc2h066*)	Transcription factor	+1.46	**+1.84**	Virulence
XM_011322702	FGSG_04580 (*fgabc1*)	ABC pleiotropic drug resistance transporter	**+1.72**	0.40	Virulence and tolerance to benalaxyl
XM_011327033	FGSG_11028	Multidrug resistance-associated protein		**+2.65**	Nivalenol biosynthesis
XM_011326203	FGSG_05898 (*fgplc1*)	Fungal phospholipase C	+1.31	**+1.66**	Development, pathogenicity, and stress responses
XM_011328541	FGSG_07133 (*gzzc230*)	Transcription factor	+1.18	**+1.72**	Virulence
XM_011329465	FGSG_07928 (*gzc2h059*)	Transcription factor	+1.29	**+1.61**	Virulence
XM_011317284	FGSG_00007	Cytochrome P450	**−3.85**	**−3.68**	DON biosynthesis
XM_011317365	FGSG_00071 (*tri1*)	Cytochrome P450	**−1.62**	−1.38	DON biosynthesis
XM_011323873	FGSG_03534 (*tri3*)	15-*O*-Acetyltransferase	**−2.99**	**−4.17**	DON biosynthesis
XM_011323872	FGSG_03535 (*tri4*)	Trichodiene oxygenase	**−3.24**	**−5.12**	DON biosynthesis
XM_011323870	FGSG_03537 (*tri5*)	Trichodiene synthase	**−2.74**	**−3.56**	DON biosynthesis
XM_011323871	FGSG_03536 (*tri6*)	Transcription factor	−1.15	**−1.65**	DON biosynthesis
XM_011323868	FGSG_03539 (*tri9*)	TRI9 protein	−1.42	**−1.84**	DON biosynthesis
XM_011323864	FGSG_03543 (*tri14*)	Mala s 1-allergenic	−**2.67**	**−3.91**	DON biosynthesis
XM_011323865	FGSG_03542	Cytochrome P450	−1.81	**−5.13**	DON biosynthesis
XM_011322312	FGSG_08196	Peptidase A4	**−3.30**	**−5.00**	Highly induced in mycotoxin-inducing media
XM_011324413	FGSG_03065 (*gzcarb*)	Phytoene dehydrogenase	−0.80	**−2.08**	Neurosporaxanthin and torulene BGC
XM_011324406	FGSG_03071	FAD-dependent oxidoreductase	−1.74	**−3.26**	Neurosporaxanthin and torulene BGC
XM_011324412	FGSG_03066 (*gzcara*)	al-2/carRA phytoene synthase	−0.77	**−1.58**	Neurosporaxanthin and torulene BGC
XM_011321137	FGSG_10460 (*fsl5*)	Enoyl reductase	1.10	**−4.27**	Fusarielin BGC
XM_011321139	FGSG_10462 (*fls3*)	Aldose 1‐epimerase	**1.54**	**−2.45**	Fusarielin BGC
XM_011321140	FGSG_10463 (*fls2*)	Esterase	**1.78**	**−2.03**	Fusarielin BGC
XM_011321141	FGSG_10464 (*fls1*)	Polyketide synthase	**1.52**	**−1.87**	Fusarielin BGC

a Significant differences (FDR < 0.05 and log_2_FC > 1.5 or <−1.5) are highlighted in boldface letters.

The other differentially expressed genes did not have a characterized functional role, but a function was predicted for some of them. In particular, among the genes upregulated during the Δ*dcl2* mutant-*B*. *cinerea* interaction, we detected 49 putatively coding for hydrolytic enzymes, 24 located in putative secondary metabolite BGCs, 22 transcription factors, 17 genes involved in RNA silencing, 15 protein kinases, and 13 MFS transporters (see Table S6C). GO enrichment analysis of upregulated genes during the Δ*dcl2* mutant-*B*. *cinerea* interactions identified terms for metabolic processes, including gene expression (GO:0010467), cellular component organization or biogenesis (GO:0071840), and RNA processing (GO:0006396) (Fig. 6B). However, GO terms oxidoreductase activity (GO:0016491), oxidation-reduction processes (GO:0055114), and polysaccharide and glucan catabolic processes (GO:0000272 and GO:0009251) were enriched for the downregulated genes (Fig. 6B).

During Δ*dcl2* mutant-F. graminearum interaction, 397 (169 upregulated and 128 downregulated) F. graminearum genes were differentially expressed, while only 97 (78 upregulated and 19 downregulated) were differentially expressed during the Δ*dcl1-*F. graminearum interaction (Fig. 6C; see also Table S6D). Totals of 47 and 16 genes were upregulated and downregulated, respectively, against both mutant strains, whereas the rest were differentially expressed only during contact with one of the mutants (Fig. 6C). Furthermore, we found 26 (9 upregulated and 17 downregulated) previously characterized F. graminearum genes that were differentially regulated during the interaction with *dcl* deletion strains compared to the WT (Table 6). The downregulated genes included several involved in deoxynivalenone, neurosporaxanthin, torulene, and fusarielin biosynthesis. Moreover, eight of the nine upregulated genes were previously characterized for having a role in F. graminearum virulence, and six of them encoded transcription factors (FgMYT3, GzHMG005, FgERB1, GzC2H066, GzZC230, and GzC2H059) (Table 6). Additionally, during the interaction with the Δ*dcl2* mutant, 14 F. graminearum CAZyme genes showed upregulation with respect to the WT, all of them predicted to encode glycoside hydrolases, whereas only 3 genes were downregulated. MFS transporters were among the DEGs as well, with five of them being upregulated while seven were downregulated (see Table S6D).

## DISCUSSION

While the Δ*dcl1* mutant had a phenotype largely similar to the WT, the Δ*dcl2* mutant displayed evident differences, including a higher number of differentially expressed genes during the interaction with the plant-pathogenic mycohosts. This number of DEGs was significantly higher than the number of genes predicted to be directly targeted from DCL2-regulated milRNAs, but it has already been observed in F. graminearum and T. atroviride how RNAi can be involved in regulating the activity of transcription factors and other regulatory elements and therefore indirectly influencing the expression of a vast array of genes and pathways (20, 69). In our data set, we could observe four *C. rosea* transcription factors downregulated in the WT during interaction with the mycohosts and putatively targeted by milRNAs downregulated in the Δ*dcl2* mutants. Among these, CRV2G00015277 and CRV2G00002266 were involved in the interaction with both the mycohosts, while CRV2G00002043 was involved only in response to *B. cinerea*. CRV2G00002266 exhibited significant sequence similarities with the PRZ1 transcription factor, known for regulating the expression of the vacuolar ATPase Ca^2+^ pump PMC1 (71). This pump shown to regulates the level of cytoplasmic Ca^2+^ by activating Ca^2+^-dependent enzymes involved in protein secretion in the nuclear envelope, endoplasmic reticulum, Golgi complex, and *trans*-Golgi/endosomal network in S. cerevisiae (71).

Furthermore, several other putative milRNA targets could have regulatory roles, including the predicted helicases CRV2G00001868 and CRV2G00004339 and the putative Rho-type GTPase activating protein CRV2G00008014. In particular, the transcript of gene CRV2G00004339, putatively targeted by milRNAs during interaction with F. graminearum, encodes a helicase of superfamily SNF2, involved in chromatin remodeling by deposition of H2A (72).

Beyond the direct action of milRNAs on targets, the deletion of *dcl1* and especially *dcl2* induced the differential expression of several secondary metabolite BGCs in *C. rosea*. The BGC containing the PKS gene *pks22*, involved in the synthesis of the antifungal compound clonorosein (50) was downregulated in the Δ*dcl2* mutant during the interaction with both mycohosts. In contrast, no difference in clonorosein A production was detected between the WT and the *dcl* mutants in the metabolome analysis. However, since the metabolome analysis was performed under *in vitro* conditions, it is possible that the *dcl2*-dependent regulation of clonorosein production is more pronounced during contact with the mycohosts. In fact, *pks22* was previously shown to be induced during interactions with *B*. *cinerea* and F. graminearum (50). The sorbicillin BGC, responsible for the yellow coloration of WT *C. rosea* colonies (50), is downregulated in the Δ*dcl2* mutant, and both sorbicillin and sorbicillinol were underproduced in the Δ*dcl2* mutant and had their biosynthesis restored in the complementation mutant in the *in vitro* trials, explaining the difference in pigmentation of the Δ*dcl2* mutant. This gene cluster was also induced during the interaction of *C. rosea* strain ACM941 with F. graminearum in the study of Demissie et al. (48). However, it is interesting that the positive regulator of the cluster, YPR1 (CRV2G00015416), is not differentially expressed in our study, whereas the transcription factor YPR2 (CRV2G00015419) is downregulated and hence coregulated with the other genes in the gene cluster in the Δ*dcl2* mutant. YPR2 is a Gal4-like transcription factor predicted to positively regulate a negative regulator of sorbicillin biosynthesis (62), and its coregulation with the biosynthetic genes suggests that the deletion of DCL2 affects the control of sorbicillin production at a currently unknown level.

Furthermore, two putatively important BGCs were specifically downregulated in the Δ*dcl2* mutant during contact with F. graminearum: these were the *pks29* BGC involved in antagonism and biocontrol (50) and the BGC with the NRPS-like CRV2G00015275 as the core enzyme. This last cluster was studied as “cluster 3” in the work of Demissie et al. (47), where it was found to be induced in *C. rosea* after exposure to the F. graminearum secretome, and it presents strong homology with the fumisoquin cluster of Aspergillus fumigatus (73). Deletion of the core NRPS-like enzyme of the cluster leads to reduced growth and sporulation in A. fumigatus (74), but fumisoquins were not produced in detectable amounts by either the WT or the Δ*dcl2* mutant in our *in vitro* analysis. Biosynthesis of the corresponding compound in *C. rosea* may be specifically triggered during contact with F. graminearum. The transcription factor CRV2G00015277, putatively targeted by DCL2-dependent novel milRNAs cro-mir-5, cro-mir-10, and cro-mir-11, is located next to the cluster and is upregulated in the Δ*dcl2* mutant. It is possible that CRV2G00015277 is a negative regulator of the cluster, targeted by milRNAs to induce the production of fumisoquins, but this hypothesis should be examined in a future study. None of these gene clusters (sorbicillin, clonoroseins, *pks29*, and fumisoquins) were downregulated in the Δ*dcl1* mutant. The reduced production of bisorbicillinol in the Δ*dcl2* mutant also suggests that the deletion might hamper this fungus’ antibacterial properties, since several bisorbicillinoids synthesized by *C. rosea* have significant antibacterial activity (75).

A further reason for the diminished capacity of the Δ*dcl2* mutant to control the plant-pathogenic mycohosts can be found in the downregulation of genes encoding enzymes involved in the degradation of the fungal cell wall. In the Δ*dcl2* mutant, between 55 and 64 glycoside hydrolase genes were downregulated compared to the WT. Among these were three GH18 chitinases (*ech37*, *ech42*, and *chiA5*) and one GH20 *N*-acetylhexosaminidase (CRV2G00012950), which were downregulated during interaction with both mycohosts. Furthermore, four genes putatively involved in cell wall degradation of F. graminearum (48) were found to be downregulated in the Δ*dcl2* mutant: these were two glycoside hydrolases of classes GH2 (CRV2G00016896) and GH114 (CRV2G00003509), as well as two metallopeptidases (CRV2G00010851 and CRV2G00011092). Interestingly, the gene *chiC1*, predicted to encode a killer toxin-like chitinase that permeabilizes the cell wall of antagonistic species to facilitate entry of toxic metabolites (76, 77), is upregulated in the Δ*dcl2* mutant. This may be explained by the fact that *chiC1* is induced by chitin (44) and that the Δ*dcl2* mutant is compromised in its ability to antagonize the mycohosts, resulting in larger amounts of chitin exposed to the Δ*dcl2* mutant.

Moreover, 17 genes upregulated during *C. rosea* response to mycohosts in the study of Nygren et al. (49) were downregulated in the Δ*dcl2* mutants in comparison with the WT upon contact with the same mycohost. Among them is a putative isotrichodermin C-15 hydroxylase (*cyp1*), a type of protein also induced during mycoparasitism in *T.* cf*. harzianum* (78), but the majority of these genes is constituted by transporters, especially MFS transporters. This group includes gene *mfs464*, suggested in the study of Nygren et al. (49) to perform an important function in the mycoparasitic attack against F. graminearum, due to its extreme induction (fold change > 693). *mfs166* and *mfs464*, downregulated in the Δ*dcl2* mutant, were found to be upregulated during the *C. rosea* response to F. graminearum in the studies of both Nygren et al. (49) and Demissie et al. (48), making their involvement in response to the mycohost very likely. The other detected differentially expressed MFS transporters are commonly involved in efflux-mediated protection against exogenous or endogenous secondary metabolites and sugar uptake, suggesting a DCL-dependent influence on this aspect of *C. rosea* mycoparasitic action. This group also includes nine genes belonging to the drug–H^+^ antiporter-2 family, which underwent a significant gene expansion during *C. rosea* evolution and has therefore a putative important role in the fungus lifestyle (79). DCL-based control of these transporters is most likely indirect because most MFS genes detected in this way are downregulated in the mutants, whereas direct targets of RNA silencing are expected to be upregulated after *dcl* deletion. Reinforcing this hypothesis, none of the MFS transporters predicted in *C. rosea* is a putative target of differentially expressed milRNAs detected in this study. Identification of several upregulated genes coding for MFS transporters used by mycohosts to tolerate harmful secondary metabolites of their own production strengthens the hypothesis that these proteins enable *C. rosea* to withstand mycohost-produced toxins during fungus-fungus interaction.

The differential expression of this vast number of genes is likely due to the 128 putative transcription factors differentially expressed in the Δ*dcl2* mutant. Among these, CRV2G00006707 is a homolog of the CCAAT-binding subunit HAP3, regulating growth and secondary metabolism in other filamentous fungi such as F. verticillioides (68, 80). This gene is downregulated in the Δ*dcl2* mutant during interaction with both mycohosts (log2 fold change [log_2_FC] of −1.6 in Cr-Bc [*C*. *rosea* + *B*. *cinerea*] and −1.3 in Cr-Fg [*C*. *rosea* + *F. graminearum*]). Another transcription factor downregulated in the Δ*dcl2* mutant was CRV2G00004759, a homolog of the filamentous growth regulator 27 (*fgr27*) of *Trichoderma lentiforme*, which is involved in adherence regulation and could have a role in reduced growth rate of the mutant (67). Moreover, two putative homologs of the sucrose utilization protein 1 (SUC1) are upregulated in the Δ*dcl2* mutant, and its upregulation is associated with a delay in mitotic and meiotic nuclear divisions in Schizosaccharomyces pombe (66).

It is possible that part of the reduced ability of the Δ*dcl2* mutant to overgrow *B. cinerea in vitro* and control F. graminearum *in vivo* comes from a cross-regulating action of *C. rosea* milRNAs targeting mycohost genes involved in the development or reduction of virulence. Specifically, three F. graminearum virulence factors were both downregulated during interaction with the WT *C. rosea* and putatively targeted by milRNAs downregulated in the Δ*dcl2* mutants. These genes included FGSG_07067, the GzZC232 transcription factor whose deletion impaired virulence in the work of Son et al. (81); FGSG_00376, the NOS1 NADH ubiquinone oxidoreductase proven to be a factor of virulence by Seong et al. (82); and FGSG_02083, the transcription factor ART1, whose deletion causes reduced starch hydrolysis and virulence, as well as the incapability of trichothecenes biosynthesis (83). Regarding *B. cinerea*, among the putative milRNA-targeted downregulated genes, there were those encoding BCIN_09g06130, the BcPls1 tetraspanin necessary for appressorium-mediated penetration into host plant leaves (84), and BCIN_16g01130, the bcpka1 catalytic subunit of the cAMP-dependent protein kinase, whose deletion affects the lesion development and leaves rot caused by the fungus (85). Two other putative targets were BcnoxA (BCIN_05g00350), a component of the *B. cinerea* NADPH oxidase complex necessary for the colonization of host tissues (86), and the MAP triple kinase BcSte11 (BCIN_03g02630), whose deletion is known to cause defects in germination, delayed vegetative growth, reduced size of conidia, lack of sclerotium formation, and loss of pathogenicity in *B. cinerea* (87). Moreover, a *B. cinerea* homolog of *Ssams2* (BCIN_08g03180) was also among the putative targets, and this gene encodes a GATA transcription factor required for appressoria formation and chromosome segregation in *S. sclerotiorum* (70).

Several other genes encoding virulence factors were found to be upregulated in the pathogenic mycohosts during the interaction with the Δ*dcl2* mutant, even if they were not among the putative targets of milRNAs. Among the F. graminearum genes upregulated during contact with the Δ*dcl2* mutant were the transcription factors MYT3, ERB1, GzHMG005, GzC2H066, GzZC230, and GzC2H059, whose disruption reduces the virulence of the pathogen (81, 88–91), as well as the phospholipase PLC1, known for its involvement in hyphal growth, conidiation, deoxynivalenol production, and virulence (92). Regarding *B. cinerea*, among the genes upregulated during contact with the Δ*dcl2* mutant, we found *nop53* and *noxR*, crucial for fungal development and virulence through the regulation of reactive oxygen species (93, 94); *frq1*, involved in circadian regulation of fungal virulence (95); and *vel1*, whose deletion affects virulence and light-dependent differentiation (96). Moreover, among the upregulated genes there was also a homolog (BCIN_14g03930) of the *S. sclerotiorum* transcription factor *SsNsd1*, necessary for pathogenicity and appressorium formation (97). Furthermore, upon contact with the Δ*dcl2* mutant, *B. cinerea* upregulated several genes encoding proteins involved in chitin and cell wall synthesis, such as Bccrh1, BcchsIV, BcchsV, BcchsVI, and BcchsVII (98–101). The upregulation of BcCHSVI and BcCHSVII is of particular interest because these proteins have a role in plant infection (101).

Genes encoding two virulence factors of F. graminearum (TRI5 and TRI6) and one of *B. cinerea* (MFSG) were downregulated during interaction with the Δ*dcl2* mutant. The gene *mfsG* is involved in *B. cinerea* virulence by providing tolerance to glucosinolate-breakdown products (102), but the *C. rosea* Δ*dcl2* mutant shows downregulation in several putative secondary metabolite clusters compared to the WT. Therefore, it is possible that the expression of *mfsG* is reduced during contact with the mutant because the lack of production of harmful compounds makes it unnecessary for the mycohost to express resistance genes. TRI5 and TRI6 are involved in the synthesis of trichothecenes (103, 104), and other genes involved in the biosynthesis of these mycotoxins are similarly downregulated during contact with the Δ*dcl2* mutant, including the genes *TRI1*, *TRI3*, *TRI4*, *TRI9*, and *TRI14* (105). This is surprising because F. graminearum overexpresses the transcription factor gene *ART1* during contact with the Δ*dcl2* mutant, and this transcription factor is known to be a positive regulator of trichothecene biosynthesis (83). The reduced ability of the Δ*dcl2* mutant to control F. graminearum may make it unnecessary for the mycohost to produce DON in high quantities, despite *ART1* overexpression. Interestingly, among the most relevant genes proven to be DON-responsive in *C. rosea* in a previous study (106), only 1 of 16 was found to be less expressed in the Δ*dcl2* mutant than in the WT during interaction with F. graminearum: a homolog of glucose repressible protein GRG1 (CRV2G00000966). Given the reduced expression of DON-biosynthesis genes by F. graminearum, the downregulation of a higher number of DON-responsive genes was expected.

Another important mycotoxin produced by F. graminearum is zearalenone, and the zearalenone hydrolase gene *zhd101* (CRV2G00011056) was found to be downregulated by the Δ*dcl2* mutant. The deletion of this gene undermines *C. rosea* mycoparasitic action against F. graminearum (107), and its downregulation is therefore a possible reason for the impaired biocontrol action of the Δ*dcl2* mutant. Another zearalenone-responsive gene, one encoding a putative bacteriorhodopsin-like protein (106), is also downregulated in the Δ*dcl2* mutant, but its role in the *C. rosea-*F. graminearum interaction is still unknown.

Interestingly, F. graminearum showed altered production of red pigment at the point of contact with the Δ*dcl2* mutant, which could plausibly be due to downregulation of genes belonging to the gene clusters of carotenoid and fusarielin (108, 109). However, the gene cluster of aurofusarins, known for their red colorations, was not differentially expressed during the interaction with the Δ*dcl2* mutant.

### Conclusions.

DCL-dependent RNA silencing plays a determinant role in the regulation of many biological processes. In the present study, the role of DCL-like enzymes was investigated for the first time in the antagonistic action of the fungus *C. rosea*. Our result show that DCL2-mediated RNAi plays a central role in regulating endogenous cellular processes involved in growth, secondary metabolite production, and antagonism toward the mycohosts, whereas the function of DCL1 is redundant except for conidium production. The observed phenotypic effect in Δ*dcl2* strains is due to the diminished production of antifungal metabolites in the mutant, as well as to downregulation of genes known to be involved in mycohost response and resistance to secondary metabolites. Identification of 11 milRNAs, which were downregulated in the Δ*dcl2* strain, and their putative endogenous gene targets, including transcription factors and chromatin remodeling proteins, indicates DCL-dependent regulation of *C. rosea* antagonistic interactions. Furthermore, we predicted putative cross-species gene targets in the mycohosts *B. cinerea* and F. graminearum previously characterized for their role in fungal virulence, posing the bases for future studies focusing on the role of DCL-dependent RNA silencing in interspecific fungal interactions.

## MATERIALS AND METHODS

### Fungal strains and culture conditions.

*C. rosea* strain IK726 WT and mutants derived from it, *B. cinerea* strain B05.10, and F. graminearum strain PH1 were used in this study. The fungal cultures were maintained on PDA (Oxoid, Cambridge, UK) medium at 25°C.

### Gene identification and phylogenetic analysis.

*C*. *rosea* strain IK726 genome version 1 (41) and version 2 (55) were screened for the presence of genes encoding DCL, AGO, and RDR by BLASTP analysis. The presence of conserved domains was analyzed using the Simple Modular Architecture Research Tool (SMART) (110), InterProScan (111), and conserved domain search (112).

Amino acid sequences of DCLs (DCL1 and DCL2), AGOs (AGO1 and AGO2), and RDRPs of several fungal species (see Table S1A) were retrieved from the UniProt and GenBank databases (113, 114). The sequences of Dicer1, Argonaute1, and RDR of the model plant Arabidopsis thaliana were retrieved from the UniProt database (113) and used as outgroups. Sequences were aligned with mafft v.7 (115) with options suggested for <200 sequences (L-INS-i), and the phylogenetic trees were generated using iqtree v.1.6.12 (116) with 1,000 bootstrap replicates and option “MFP” to find the best substitution model. Figtree v.1.4.4 (117) was used to visualize the trees.

### Construction of deletion vector, transformation, and mutant validation.

The ∼1-kb 5′-flank and 3′-flank regions of *dcl1* and *dcl2* were amplified from genomic DNA of *C*. *rosea* using gene-specific primer pairs (see Table S1B), as indicated in Fig. S1 (53). Gateway cloning system (Invitrogen, Carlsbad, CA) was used to generate entry clones of the purified 5′-flank and 3′-flank PCR fragments as described by the manufacturer (Invitrogen, Carlsbad, CA). The hygromycin resistance cassette (hygB) generated during our previous studies (43, 118) from pCT74 vector, as well as a Geneticin resistance cassette generated as a PCR product from the pUG6 vector (119), were used. A three-fragment multisite gateway LR recombination reaction was performed using the entry plasmids of respective fragments and destination vector pPm43GW (120) to generate the deletion vectors. Complementation cassettes for *dcl1* and *dcl2* were constructed by PCR amplification of the full-length sequence of *dcl1* and *dcl2*, including ∼800-bp upstream and ∼500-bp downstream regions from genomic DNA of *C. rosea* WT using gene-specific primers (see Table S1B). The amplified DNA fragments were purified and integrated into destination vector pPm43GW using two-fragment gateway cloning technology to generate complementation vectors.

Agrobacterium tumefaciens-mediated transformation was performed based on a previous protocol for *C. rosea* (43, 121). Transformed strains were selected on plates containing either hygromycin for gene deletion or Geneticin for complementation. Validation of homologous integration of the deletion cassettes in putative transformants were performed using a PCR screening approach with primer combinations targeting the hygB cassette and sequences flanking the deletion cassettes (see Fig. S1), as described previously (45, 122). PCR-positive transformants were tested for mitotic stability and then purified by two rounds of single-spore isolation (118). To determine the transcription of *dcl1* and *dcl2* in the WT, deletion, and complementation strains, total RNA from the respective strains were isolated (Qiagen, Hilden, Germany). After DNase I treatment, according to the manufacturer’s instructions (Merck, Kenilworth, NJ) reverse transcription-PCR (RT-PCR) was performed using RevertAid premium reverse transcriptase (Fermentas, St. Leon-Rot, Germany) and gene-specific primer pairs (see Table S1B).

### Phenotypic analyses.

Phenotypic analyses experiments were performed with *C*. *rosea* WT, deletion strains *dcl1* (Δ*dcl1*) and *dcl2* (Δ*dcl2*), and their respective Δ*dcl1*+ and Δ*dcl2*+ complemented strains. Each experiment was repeated twice with similar results.

The growth rate, colony morphology, and conidium production were analyzed in four biological replicates as described previously (43). To analyze mycelial biomass, agar plugs of *C*. *rosea* strains were inoculated in 50-ml conical flasks with 20 ml of PDB (Oxoid, Cambridge, UK), followed by incubation at 25°C under constant shaking (100 rpm). Biomass production was determined by measuring the mycelial dry weight 5 days postinoculation. The antagonistic behavior against *B*. *cinerea* and F. graminearum was tested using an *in vitro* plate confrontation assay on PDA medium, as described previously (51). The growth of *B*. *cinerea* and F. graminearum was measured daily until their mycelial fronts touched the *C*. *rosea* mycelial front. The experiments were performed in four biological replicates. The biocontrol ability of *C. rosea* strains against F. graminearum was evaluated in a fusarium foot rot assay, as described previously (123). In brief, surface sterilized wheat seeds were treated with *C. rosea* conidia (1 × 10^7^ conidia/ml) in sterile water, sown in moistened sand, and kept in a growth chamber after pathogen inoculation (51). Plants were harvested 3 weeks postinoculation, and disease symptoms were scored on scale of 0 to 4, as described previously (51, 123). The experiment was performed in five biological replicates with 15 plants in each replicate.

### Statistical analysis.

ANOVA was performed on phenotype data using the general linear model approach implemented in Statistica version 16 (TIBCO Software, Inc., Palo Alto, CA). Pairwise comparisons were made using the Tukey-Kramer method at a 95% significance level.

### Metabolite analysis.

An agar plug of *C*. *rosea* strains was inoculated on PDA (Oxoid) and allowed to grow for 10 days at 25°C. Agar plugs, together with mycelia, were harvested from the centers of plates using 50-ml Falcon tubes (53). The mycelial plug was sonicated for 15 min in 20 ml of methanol, and then 1 ml of extract was transferred to a 1.5-ml centrifuge tube for centrifugation at 10,000 × *g* for 5 min. Supernatants were collected and then analyzed by UHPLC-MS on a reversed-phase column (2.1 × 50 mm, 1.5 μm; Accucore Vanquish; Thermo Scientific, Waltham, MA) using a gradient of acetonitrile (MeCN) in water, both with 0.2% formic acid (10 to 95% MeCN in 3 min and 95% MeCN for 1.2 min, at 0.9 ml min^−1^). The MS was operated in positive mode with scanning of *m/z* 50 to 1,500, and the mass spectra were calibrated against sodium formate clusters using the Compass DataAnalysis 4.3 software (Bruker Daltonics, Bremen, Germany) that was also used for general data analysis. UHPLC-MS/MS was run with the same instrument, column, and UHPLC conditions, using the auto-MS/MS function (1+ precursor ions, *m/z* 50 to 1,500, with ramped fragmentation energies of 20/30/35 eV for *m/z* 200/500/1,000). The UHPLC-MS data were converted to mzXML format using DataAnalysis 4.3, and ion chromatogram peak picking in the range 5 to 200 s was performed using the program XCMS in software environment R using the centWave method (peak width, 3 to 20 s; *m/z* tolerance, 5 ppm; noise, 1,000) (124, 125). XCMS was used for subsequent peak grouping and missing peak filling. For each sample, the resulting molecular feature peak areas were normalized against the sum of peak areas, and the resulting relative peak areas were 10 logarithmized. The data were used for PCA, and ANOVA was used to evaluate significant differences in concentrations between strains. Tentative compound identification was done by comparing high-resolution mass spectrometry data on fungal compounds from the databases Antibase and combined chemical dictionary. The identity of the tentatively identified compounds was further corroborated by analysis of MS/MS data. The experiment was performed in five biological replicates.

### Dual culture interaction experiment for sRNA and transcriptome sequencing.

An agar plug of *C*. *rosea* strains was inoculated at edge of a 9-cm-diameter PDA (Merck, Kenilworth, NJ) petri plate covered with a Durapore membrane filter (Merck) for easy harvest of mycelia. The mycohost fungi *B. cinerea* or F. graminearum were inoculated at opposite side of the plate (43). Due to different mycelial growth rates, *C*. *rosea* was inoculated 7 days prior to the inoculation of F. graminearum or *B*. *cinerea*. The mycelial front (5 mm) of *C*. *rosea* was harvested together with the mycelial front (5 mm) of *B*. *cinerea* (Cr-Bc) or F. graminearum (Cr-Fg) at the hyphal contact stage of interactions (see Fig. S2A) and snap-frozen in liquid nitrogen. The experiment was performed in three biological replicates.

### RNA extraction, library preparation, and sequencing.

Total RNA was extracted using the mirVana miRNA isolation kit according to the manufacturer’s protocol (Invitrogen, Waltham, MA). The RNA quality was analyzed using a 2100 Bioanalyzer Instrument (Agilent Technologies, Santa Clara, CA) and concentration was measured using a Qubit fluorometer (Life Technologies, Carlsbad, CA). For sRNA and mRNA sequencing, the total RNA was sent for library preparation and paired-end sequencing at the National Genomics Infrastructure (NGI), Stockholm, Sweden. The sRNA library was generated using TruSeq small RNA kit (Illumina, San Diego, CA), while the mRNA library was generated using a TruSeq Stranded mRNA Poly(A) selection kit (Illumina, San Diego, CA). The sRNA and mRNA libraries were sequenced on a NovaSeq SP flow cell with a 2 × 50-bp reads and NovaSeqXp workflow in S4 mode flow cell with 2 × 151 setup, respectively, using Illumina NovaSeq6000 equipment at NGI Stockholm. The Bcl to FastQ conversion was performed using bcl2fastq_v2.19.1.403 from the CASAVA software suite (126). The quality scale used was Sanger/phred33/Illumina 1.8+.

### (i) Functional annotation of genomes.

The predicted proteomes of *C. rosea* strain IK726, *B. cinerea* strain B05.10 (ASM14353v4), and F. graminearum strain PH-1 (ASM24013v3) were annotated through BLAST2GO v.5.2.5 (127) and InterProScan v.5.46-81.0 (111) with default parameters to identify transcription factors. Secondary metabolite clusters were predicted through antiSMASH v.5.0 (128), while predicted enzymes involved in the metabolism of carbohydrates (CAZymes) were identified using the dbCAN2 meta-server (129). The amino acid sequences of *B*. *cinerea* and F. graminearum were compared to the PHI-base database using BLAST (130) with a minimum of 80% in both identity and query coverage. All identified matches described in the PHI-base annotation by the keywords “reduced virulence” or “loss of pathogenicity” were considered to be potential virulence factors.

### (ii) Differential expression and GO enrichment analyses.

Reads were trimmed with bbduk v.38.86 (131) with the following options: bbduk.sh in1=read1.fastq in2=read2.fastq out1=read1_clean.fastq out2=read2_clean.fastq ref=reference.fa ktrim=r k =23 mink=11 hdist=1 tpe tbo qtrim=r trimq=10. Successful cleaning and adapter removal was verified with fastqc v. 0.11.9 (https://www.bioinformatics.babraham.ac.uk/projects/fastqc/). Since all the samples represented the interaction of two organisms, the genome of *C. rosea* was concatenated with the one of either *B. cinerea* or F. graminearum, creating two “combined genomes” (Cr-Fg and Cr-Bc), and the same was done with the annotations in .gff format. Reads from the *C. rosea*-*B. cinerea* interaction were aligned to the Cr-Bc genome, whereas reads from the *C. rosea*-F. graminearum interaction were aligned to the Cr-Fg. The chosen aligner was STAR v.2.7.5c (132), with default options, and the count tables were then generated through featureCounts v.2.0.1 (133). Finally, the differential expression analysis was done with DESeq2 v.1.28.1 (134), where an FDR of <0.05 in combination with a log_2_FC of >1.5 or <−1.5 was considered to define differentially expressed genes (DEGs). Enrichment in gene ontology (GO) terms of DEGs was determined through Fisher tests integrated in the BLAST2GO suite, with an FDR threshold of 0.05.

### (iii) Mapping of sRNAs.

sRNA reads were trimmed with bbduk v.38.86 (131) with the same options used for mRNA read trimming, and successful cleaning and adapter removal was verified with fastqc v.0.11.9. The program SortMeRna v.4.2.0 (135) was used to remove structural sRNA (rRNA, tRNA, snRNA, and snoRNA) from the reads, and sequences within the length range of 18 to 32 bp were isolated with the command reformat.sh of the BBTools suite (131). The database of structural RNAs used for SortMeRna consisted in the rRNA sequences of the SILVA database (136), while snRNA, tRNA, and snoRNA sequences were downloaded from the NRDR database (137). After filtering, the sRNA reads were mapped to the Cr-Bc and Cr-Fg genomes with STAR, with the following options recommended for sRNA mapping: STAR –genomeDir index/–readFilesIn read1.fq read2.fq –outFileNamePrefix prefix –outFilterMismatchNoverLmax 0.05 –outFilterMatchNmin 16 –outFilterScoreMinOverLread 0 –outFilterMatchNminOverLread 0 –alignIntronMax 1 –alignEndsType EndToEnd. For the STAR default option, reads with good mapping results on more than 20 different loci were considered “not mapped.”

Untranslated regions (UTRs) and introns were added to the .gff files of the genomes through “add_utrs_to_gff” (https://github.com/dpryan79/Answers/tree/master/bioinfoSE_3181) and GenomeTools with the “-addintrons” option (138), respectively. Promoters were also added through an *ad hoc* Python script (https://github.com/EdoardoPiombo/promoter_extractor), considering promoters to be composed of the first 1,000 bases upstream of a gene or of all the bases until the end of the precedent gene. Introns, promoters, and UTRs were all considered when featureCounts was used to generate the count tables.

### (iv) Prediction of miRNA-like RNAs and subsequent analyses.

Putative milRNAs were predicted with mirdeep2 v.2.0.1.2 (139). The miRbase database (140), as well as all the fungal milRNA sequences from RNAcentral (141), were used to provide reference sequences from other species. To ensure the novelty of newly detected milRNAs, BLAST was used to compare them to the fungal milRNAs identified in several other studies, plus all the fungal milRNAs available in RNAcentral, requiring 95% minimum identity and query coverage (25, 33, 141–145). Nonstructural sRNA reads, previously mapped to the genomes with STAR, were counted with featureCounts, and the differentially expressed milRNAs were identified with DESeq2, with the same thresholds used for DEG analysis.

The sRNA_toolbox (146) was used to predict putative targets for the identified milRNAs. The prediction was carried out with the animal-based tools PITA, Miranda, TargetSpy (147–149), and simple seed analysis and with the plant-based tools psRobot, TAPIR FASTA, and TAPIR RNAhybrid (150, 151). Target-milRNA couples identified by at least three animal-based tools or two plant-based ones were retained for the following analyses. Predicted targets were retained only when they were significantly expressed (FDR < 0.05) with a log_2_FC >1.0 opposite to the milRNA. Putative targets of downregulated milRNAs were therefore considered only when they were overexpressed. The predicted targets present in double copy in their genome were then removed from the analysis. Repetitive elements in the genome of *C. rosea* were predicted according to the guidelines for basic repeat library construction (http://weatherby.genetics.utah.edu/MAKER/wiki/index.php/Repeat_Library_Construction-Basic), using all fungal transposons in RepetDB as known transposons (152), and putative milRNA targets within 700 bp from any *C. rosea* transposon were removed from the analysis.

### (v) Validation of milRNA-expression through stem-loop RT-qPCR.

milRNA specific stem-loop RT-qPCR primers (see Table S1B) were designed as described previously (153). Stem-loop RT primers (1 μM) were denatured at 65°C for 5 min and immediately transferred to ice. For each milRNA RT reaction, a “no RNA” master mix was prepared with 0.5 μl of 10 mM dNTP (Thermo Scientific, Waltham, MA), 5× SSIV buffer, 2 μl of 0.1 M dithiothreitol, 0.1 μl of RiboLock RNase inhibitor (40 U/μl), 0.25 μl of SSIII reverse transcriptase (Invitrogen, Waltham, MA), 1 μl of denatured stem-loop RT primer, and 1 μl of 5 μM reverse primer of *C. rosea* actin (*act*) reference gene. Next, 10 ng of RNA template used for next-generation sequencing analysis was added into respective reactions. The tubes were then incubated in a thermal cycler at 16°C for 30 min, followed by 60 cycles of pulsed RT at 30°C for 30 s, 42°C for 30 s, and 50°C for 1 s and then enzyme inactivation at 85°C for 5 min. Quantitative PCR was performed using DyNAmo Flash SYBR green kit (Thermo Scientific, Waltham, MA) according to the manufacturer’s instructions. The *C_T_* values of milRNA were normalized to that of *act* to be used for quantification using the ΔΔ*C_T_* method (154).

### Data availability.

The raw sequencing data were submitted to ENA in under BioProject accession number PRJEB43636. This project contains both the transcriptome and the sRNA sequencing data for each of the samples.
