# Supplementary material for: Role of Dicer-Dependent RNA Interference in Regulating Mycoparasitic Interactions
Source: Microbiol Spectr. 2021 Sep 22;9(2):e01099-21. doi: 10.1128/Spectrum.01099-21 (PMC8557909; doi:10.1128/Spectrum.01099-21)
Supplement: SUPPLEMENTAL FILE 6 — Supplemental material. Download SPECTRUM01099-21_Supp_6_seq15.pdf, PDF file, 1.7 MB [file spectrum01099-21_supp_6_seq15.pdf]

**Supplemental materials**

**Table S1A:** Protein ID of the putative argonauts, dicer like proteins and RNA dependent RNA polymerases proteins used in phylogenetic analyses.

**Table S1B:** List of primers used in this study.

**Table S1C:** Characteristics of the putative argonauts dicer like proteins and RNA dependent RNA polymerases proteins in *Clonostachys rosea.*

**Table S2:** Secondary metabolite analysis of *C. rosea* WT, Δ*dcl1* and Δ*dcl2*, and Δ*dcl1*+ and Δ*dcl2***+** strains by UHPLC-MS and -MSMS.

**Table S3:** Results of STAR and featureCounts based mapping of mRNA reads. The reads were mapped to the concatenated genomes of *C. rosea* and the mycohot interacting with it, which was either *B. cinerea* or *F. graminearum*, depending on the sample.

**Table S4:** Differentially expressed *C. rosea* genes during the interspecies interactions.

**Table S5A:** Expression and annotation of MFS transporters, secondary metabolite gene clusters, glycoside hydrolases, ABC transporters and transcription factors differentially expressed in this study.

**Table S5B:** *C. rosea* genes associated with gene silencing machinery and chromatin modification.

**Table S6A:** Results of STAR and featureCounts based mapping of sRNA reads. The reads were mapped to the concatenated genomes of *C. rosea* and the mycohot interacting with it, which was either *B. cinerea* or *F. graminearum*, depending on the sample.

**Table S6B:** Sequence, location, and expression values of milRNAs detected in the study.

**Table S6C:** Annotation and expression level of mycohost genes overexpressed during contact between *C. rosea* Δ*dcl2* mutant and the pathogens, putatively targeted by *C. rosea* milRNAs underexpressed in the same interaction.

**Table S6D:** Differentially expressed *B. cinerea* and *F. graminearum* genes during the interaction with *C. rosea* WT, Δ*dcl1* and Δ*dcl2* strains.

**Figure S1:** Generation and validation of gene deletion and complementation strains.

**Figure S2 (A):** Setup for dual culture interactions. (**B**): Diagrammatic representation of protein domains predicted by Simple Modular Architecture Research Tool (SMART) and Conserved Domain Database (CDD) within Dicer-like (I), argonaut (II) and RNA-dependent RNA polymerase (III) proteins. (**C-E**): Phylogenetic trees presenting the evolutionary relationship among dicer like proteins (**C**), argonauts (**D**) and RNA dependent RNA polymerases (**E**) of several fungal plant pathogens. The trees were generated with iqtree v.1.6.12 and visualized with figtree v.1.4.4.

**Figure S3A:** Phenotypic characterization of *C. rosea* strains.

**Figure S3B:** Heatmaps showing all compounds significantly underproduced in the Δ*dcl1*/Δ*dcl2* strains compared to WT and significantly overproduced in the Δ*dcl1+*/Δ*dcl2+* strains compared to the Δ*dcl1*/Δ*dcl2* strains. (**I**) WT/Δ*dcl1*/Δ*dcl1+*. (**II**) WT/Δ*dcl2*/Δ*dcl2+*. Dark red: high concentration. White: low concentration.

**Figure S3C**: Tentative identification of selected sorbicillin type compounds by UHPLC-MSMS. (**I**) Selected MSMS spectra. (**II-VI**) Proposed formation of observed fragment ions for oxosorbicillinol, sorbicillinol, epoxysorbicillinol, sorbicillin and bisvertinolone, respectively.

**Table S3**: Results of STAR and featureCounts based mapping of mRNA reads. The reads were mapped to the concatenated genomes of *C. rosea* and the mycohost interacting with it, which was either *B. cinerea* or *F. graminearum*, depending on the sample.

| Sample | Clean reads | Assigned | Cr | Bc or Fg | Unassigned | Multi maping | No Features | Ambiguity | Total |
|---|---|---|---|---|---|---|---|---|---|
| WT-Bc_1 | 21398418 | 48.06% | 25.40% | 22.66% | 21.19% | 9.23% | 16.67% | 4.84% | 100.00% |
| WT-Bc_2 | 16284375 | 46.79% | 25.30% | 21.49% | 20.20% | 11.46% | 16.66% | 4.90% | 100.01% |
| Δ*dcl1*-Bc_1 | 17242507 | 54.37% | 32.61% | 21.76% | 17.24% | 9.03% | 14.26% | 5.10% | 100.00% |
| Δ*dcl1*-Bc_2 | 22615484 | 51.34% | 29.13% | 22.21% | 18.19% | 9.58% | 15.88% | 5.02% | 100.00% |
| Δ*dcl1*-Bc_3 | 21983607 | 52.07% | 30.58% | 21.48% | 18.15% | 9.56% | 15.27% | 4.96% | 100.01% |
| Δ*dcl2*-Bc_1 | 25068347 | 46.86% | 15.85% | 31.01% | 19.36% | 8.14% | 19.48% | 6.16% | 100.00% |
| Δ*dcl2*-Bc_2 | 28703528 | 46.59% | 16.04% | 30.55% | 20.15% | 7.73% | 19.58% | 5.95% | 100.01% |
| Δ*dcl2*-Bc_3 | 17963847 | 45.67% | 15.08% | 30.58% | 18.81% | 8.94% | 20.43% | 6.16% | 100.01% |
| WT-Fg_1 | 17968620 | 81.93% | 61.79% | 20.14% | 0.89% | 4.47% | 9.86% | 2.86% | 100.01% |
| WT-Fg_2 | 17616681 | 81.69% | 58.35% | 23.35% | 0.76% | 4.54% | 10.05% | 2.96% | 100.00% |
| WT-Fg_3 | 19589685 | 80.93% | 60.98% | 19.95% | 0.76% | 4.87% | 10.43% | 3.01% | 100.01% |
| Δ*Δdcl1*-Fg_1 | 18408543 | 79.61% | 55.66% | 23.95% | 1.06% | 6.26% | 9.76% | 3.31% | 100.00% |
| Δ*dcl1*-Fg_2 | 23830764 | 79.93% | 54.96% | 24.96% | 1.16% | 6.21% | 9.43% | 3.28% | 100.00% |
| Δ*dcl1*-Fg_3 | 15277742 | 79.66% | 56.85% | 22.81% | 1.35% | 6.39% | 9.35% | 3.24% | 99.99% |
| Δ*dcl2*-Fg_1 | 21255917 | 81.60% | 58.08% | 23.52% | 1.69% | 5.16% | 8.72% | 2.82% | 99.99% |
| Δ*dcl2*-Fg_2 | 21401041 | 82.12% | 55.64% | 26.48% | 1.25% | 5.13% | 8.86% | 2.64% | 100.00% |
| Δ*dcl2*-Fg_3 | 21443308 | 82.42% | 58.98% | 23.44% | 1.28% | 5.04% | 8.62% | 2.64% | 100.00% |

Abbreviations: Cr, *Clonostachys rosea*; Bc, *Botrytis cinerea*; Fg, *Fusarium graminearum*.

**Figure S1: Generation and validation of *dcl1* and *dcl2* deletion and complementation strains**

Single *dcl1* and *dcl2* deletion mutants were generated by exchanging *dcl1* and *dcl2* with the hygromycin resistance gene selection cassette (hygB) and were confirmed by PCR using primers located within the hygB cassette together with primers located upstream or downstream of the *dcl*1 or *dcl*2 gene deletion construct (**Figure S1A**). The expected size of PCR fragments were amplified in Δ*dcl1 and* Δ*dcl2*, while no amplification was observed in the WT (**Figure S1B, S1C**). Furthermore, RT-PCR using primers specific to the *dcl1* and *dcl2* gene demonstrated the complete loss of *dcl1* and *dcl2* transcript in respective gene deletion mutants, while expression of *dcl1* and *dcl2* were detected in the WT **(Figure S1D**).

The Δ*dcl1 and* Δ*dcl2* strains were complemented with *dcl1* and *dcl2*, respectively. Successful integration of the complementation cassette in mitotically stable mutants was confirmed by PCR amplification of geneticin resistant selection cassette. RT-PCR from randomly selected geneticin positive Δ*dcl1 and* Δ*dcl2* strains using *dcl1- and dcl2*- specific primer pairs, demonstrated restored *dcl1* or *dcl2* transcription in Δ*dcl1 and* Δ*dcl2* complemented (Δ*dcl1*+; Δ*dcl2*+) strains, respectively, while no transcripts were detected in the parental deletion strains (**Figure S1E**).

**Figure S1 A**

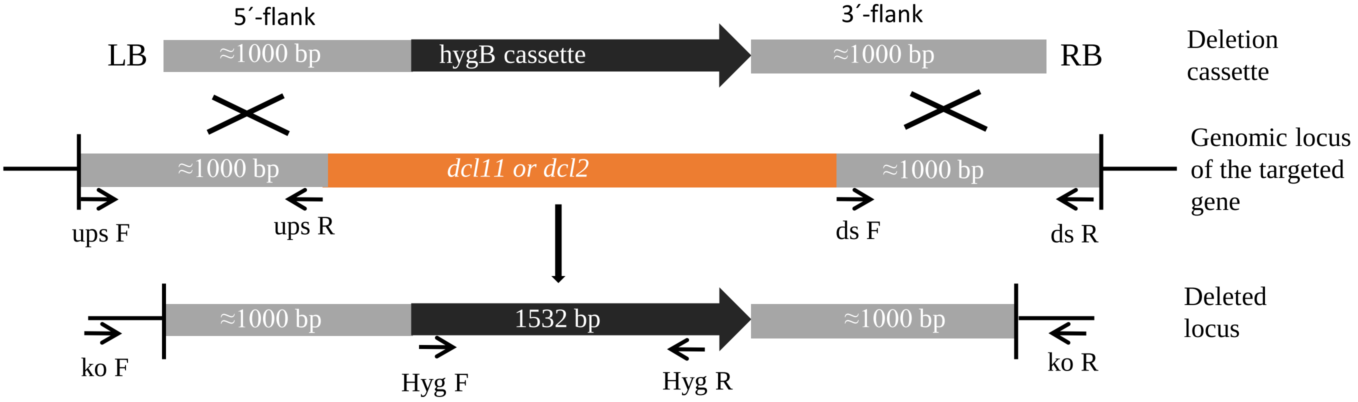


**Figure S1 A:** Organisation of Δ*dcl1* or Δ*dcl2* locus in *C. rosea* WT and mutant strains. The coding region of respective gene was replaced by hygB cassette by homologous recombination resulting in generation of deletion strains. The arrow heads indicate the location of primers used to construct the deletion cassette and analysis of mutants using PCR. Abbreviations: LB, left border; RB, right border.

**Figure S1 B**

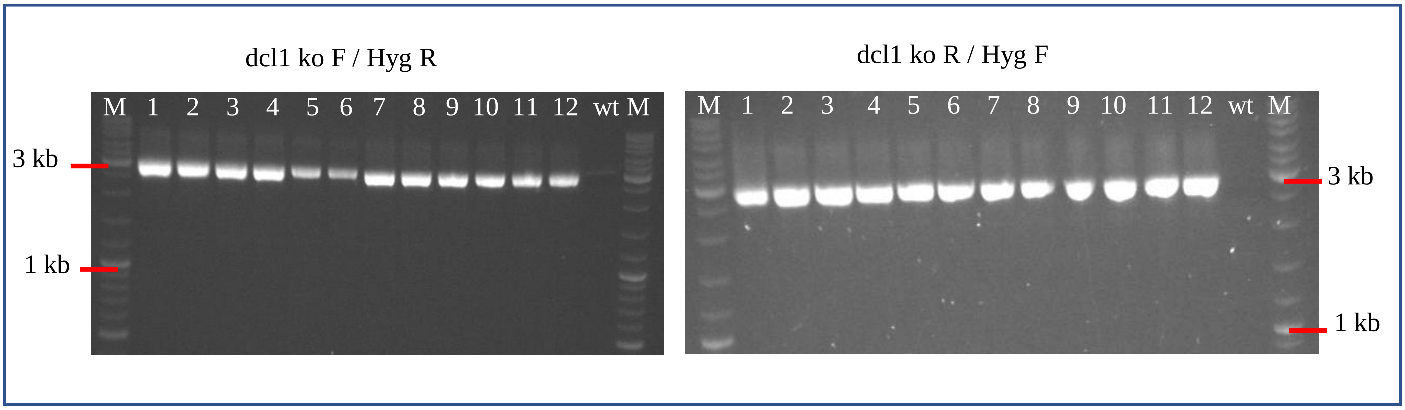


**Figure S1 C**

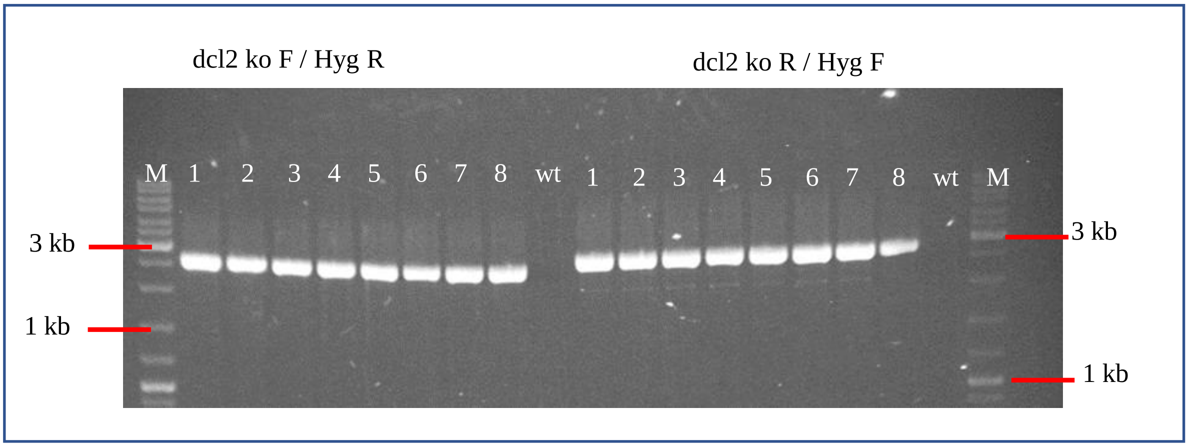


**Figure S1 D**

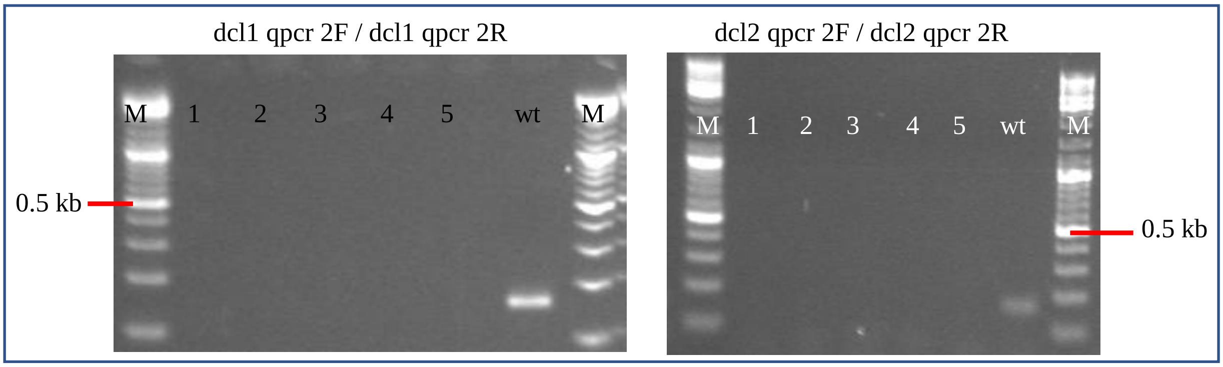


**Figure S1 E**

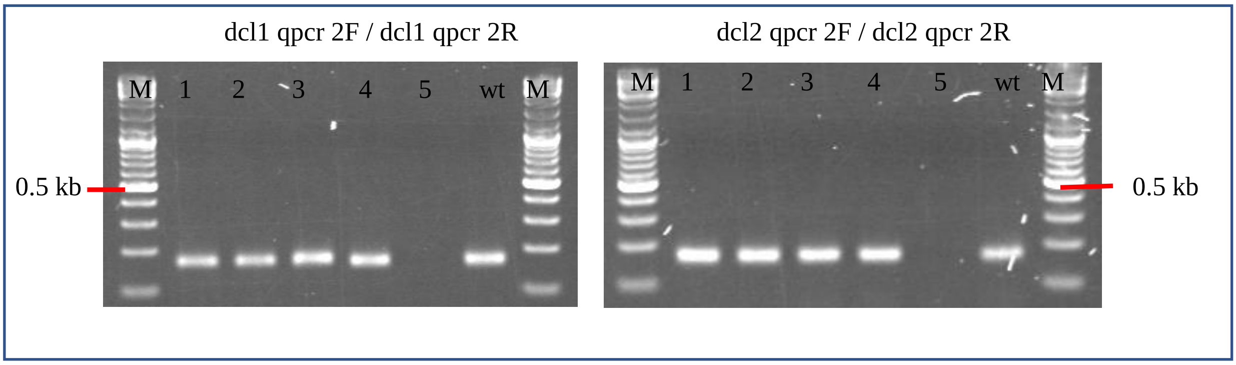

**Figure S1 B:** PCR verification of *dcl1* deletion strains using primers located in the hygB cassette (Hyg F / Hyg R) in combination with primers located upstream and downstream from the deletion cassette (dcl1 ko F / dcl1 ko R) . A PCR product of ~2.8 kb using primers dcl1 ko F / Hyg R, and dcl1 ko R / Hyg F were expected from a correct gene replacement. M, gene ruler DNA ladder mix; 1-12, independent Δ*dcl1* strains; wt, *C. rosea* wild type strain. Primer combinations used for PCR are given above the images.

**Figure S1 C:** PCR verification of *dcl2* deletion srains using primers located in the hygB cassette (Hyg F / Hyg R) in combination with primers located upstream and downstream from the deletion cassette (dcl2 ko F / dcl2 ko R). A PCR product of ~2.8 kb using primers dcl2 ko F / Hyg R, and dcl2 ko R / Hyg F were expected from a correct gene replacement. M, gene ruler DNA ladder mix; 1-8, independent Δ*dcl2* strains; wt, *C. rosea* wild type strain. Primer combinations used for PCR are given above the images.

**Figure S1 D:** RT-PCR analysis of *dcl1* and *dcl2* expression in WT and deletion strains using *dcl1* and *dcl2* specific primers dcl1 qpcr 2F / dcl1 qpcr 2R and dcl2 qpcr 2F / dcl2 qpcr 2R, respectively. A PCR product of 143 bp using primers dcl1 qpcr 2F / dcl1 qpcr 2R was expected from the WT strains. A PCR product of 151 bp using primers dcl2 qpcr 2F / dcl2 qpcr 2R was expected from the WT strains. M, gene ruler DNA ladder mix; 1-5, independent Δ*dcl1* or Δ*dcl2* mutants; wt, *C. rosea* wild type strain. Primer combinations used for RT-PCR are given above the images

**Figure S1 E:** Validation of complementation strains. RT-PCR analysis of *dcl1* expression in Δ*dcl1* complemented (Δ*dcl1+*), *dcl2* complemented (Δ*dcl2+*), and Δ*dcl1*, and Δ*dcl2* strains, using *dcl11* and *dcl2* specific primers. A PCR product of 143 bp using primers dcl1 qpcr 2F / dcl1 qpcr 2R was expected from Δ*dcl1+* and WT strains. A PCR product of 151 bp using primers dcl2 qpcr 2F / dcl2 qpcr 2R was expected from Δ*dcl2+* and WT strains. M, gene ruler DNA ladder mix; 1-4, independent complemented strains; 5, deletion strain; wt, *C. rosea* wild type strain. Primer combinations used for RT-PCR are given above the images

**Figure S2A**

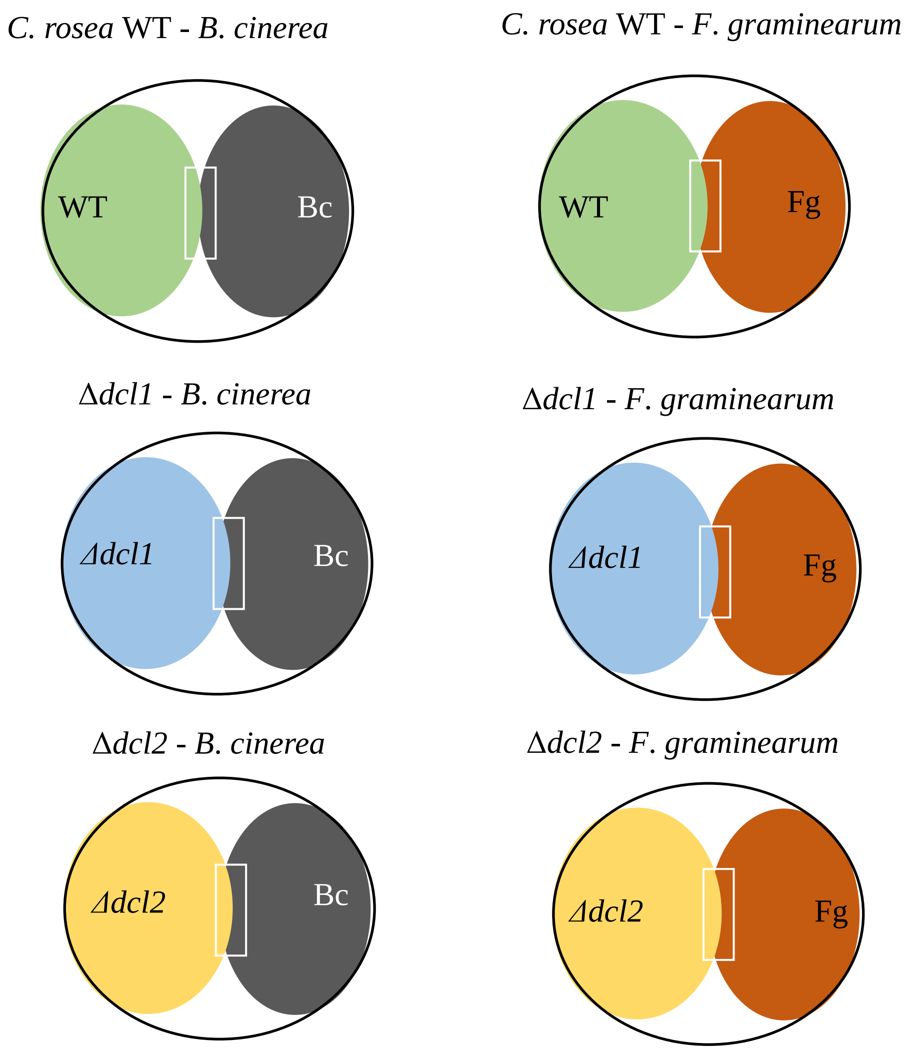


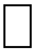 Denotes harvesting area for total RNA extraction.

WT, *Clonostachys rosea* wild type strain;

Bc, *Botrytis cinerea*;

Fg, *Fusarium graminearum*

**Figure S2A:** Dual culture interactions setup for small RNAs and mRNA sequencing experiment

**Figure S2B**

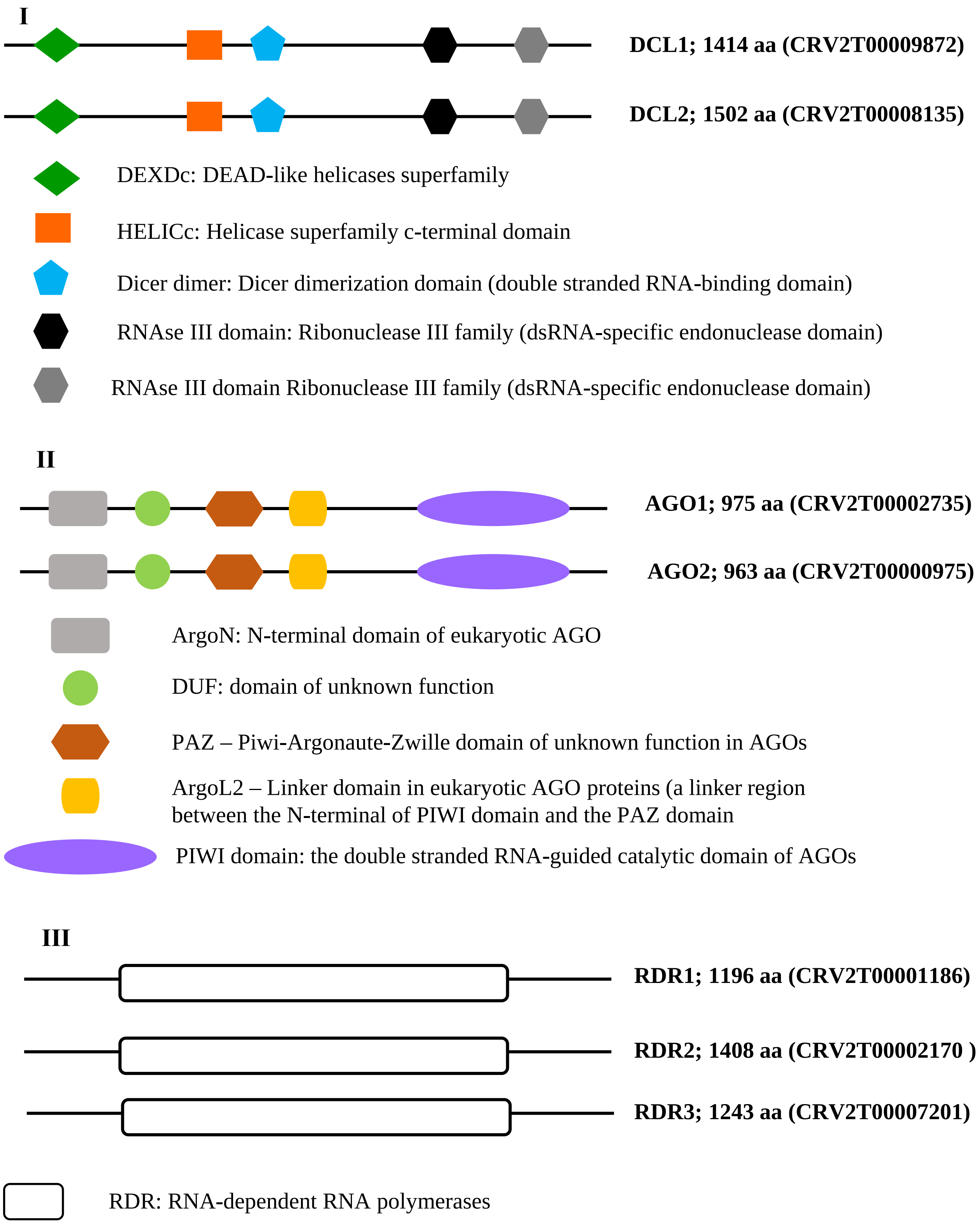


**Figure S2B:** Diagrammatic representation of protein domains predicted by Simple Modular Architecture Research Tool (SMART) and Conserved Domain Database (CDD) within Dicer-like (A) argonout (B) and RNA-dependent RNA polymerase (C) proteins.

**Figure S2C**

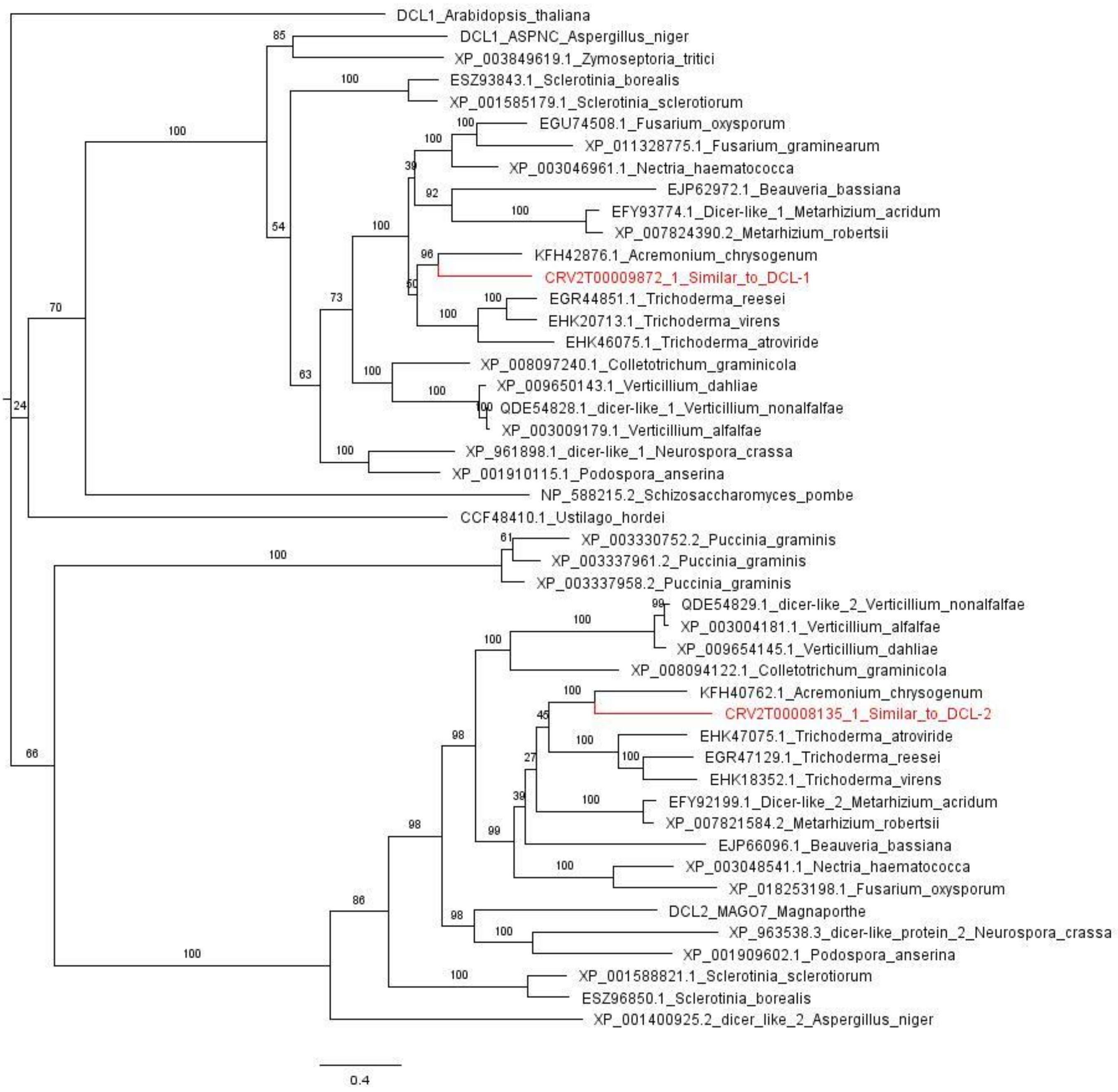
DCL1_Arabidopsis_thaliana
DCL1_ASPNC_Aspergillus_niger
XP_003849619.1_Zymoseptoria_tritici
ESZ93843.1_Sclerotinia_borealis
XP_001585179.1_Sclerotinia_sclerotiorum
EGU74508.1_Fusarium_oxysporum
XP_011328775.1_Fusarium_graminearum
XP_003046961.1_Nectria_haematococca
EJP62972.1_Beauveria_bassiana
EFY93774.1_Dicer-like_1_Metarhizium_acridum
XP_007824390.2_Metarhizium_robertsii
KFH42876.1_Acremonium_chrysogenum
CRV2T00009872_1_Similar_to_DCL-1
EGR44851.1_Trichoderma_reesei
EHK20713.1_Trichoderma_virens
EHK46075.1_Trichoderma_atroviride
XP_008097240.1_Colletotrichum_graminicola
XP_009650143.1_Verticillium_dahliae
QDE54828.1_dicer-like_1_Verticillium_nonalfalfae
XP_003009179.1_Verticillium_alfalfae
XP_961898.1_dicer-like_1_Neurospora_crassa
XP_001910115.1_Podospora_anserina
NP_588215.2_Schizosaccharomyces_pombe
CCF48410.1_Ustilago_hordei
XP_003330752.2_Puccinia_graminis
XP_003337961.2_Puccinia_graminis
XP_003337958.2_Puccinia_graminis
QDE54829.1_dicer-like_2_Verticillium_nonalfalfae
XP_003004181.1_Verticillium_alfalfae
XP_009654145.1_Verticillium_dahliae
XP_008094122.1_Colletotrichum_graminicola
KFH40762.1_Acremonium_chrysogenum
CRV2T00008135_1_Similar_to_DCL-2
EHK47075.1_Trichoderma_atroviride
EGR47129.1_Trichoderma_reesei
EHK18352.1_Trichoderma_virens
EFY92199.1_Dicer-like_2_Metarhizium_acridum
XP_007821584.2_Metarhizium_robertsii
EJP66096.1_Beauveria_bassiana
XP_003048541.1_Nectria_haematococca
XP_018253198.1_Fusarium_oxysporum
DCL2_MAGO7_Magnaporthe
XP_963538.3_dicer-like_protein_2_Neurospora_crassa
XP_001909602.1_Podospora_anserina
XP_001588821.1_Sclerotinia_sclerotiorum
ESZ96850.1_Sclerotinia_borealis
XP_001400925.2_dicer_like_2_Aspergillus_niger
0.4

**Figure S2D**

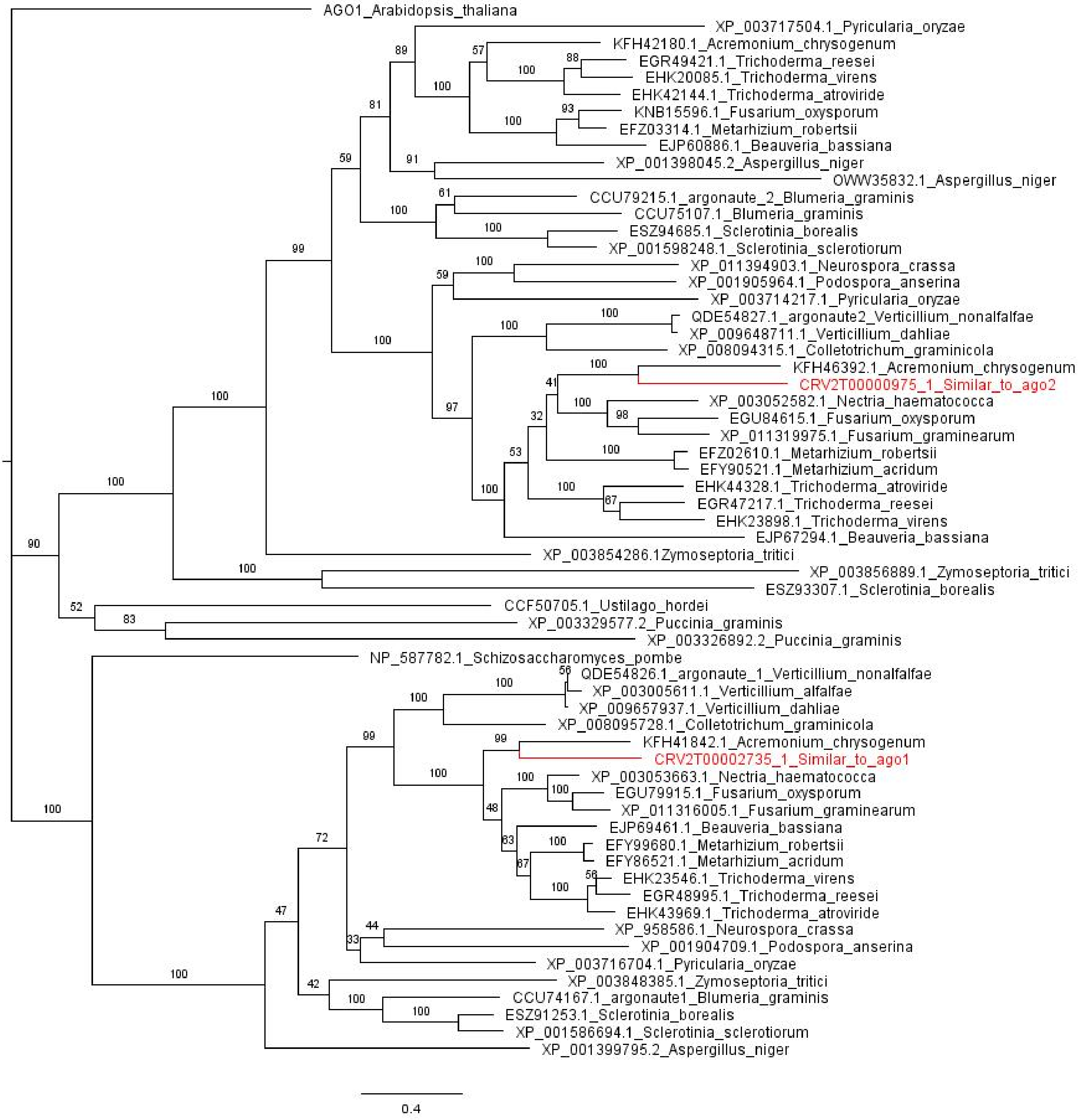
AGO1_Arabidopsis_thaliana
XP_003717504.1_Pyricularia_oryzae
KFH42180.1_Acremonium_chrysogenum
EGR49421.1_Trichoderma_reesei
EHK20085.1_Trichoderma_virens
EHK42144.1_Trichoderma_atroviride
KNB15596.1_Fusarium_oxysporum
EFZ03314.1_Metarhizium_robertsii
EJP60886.1_Beauveria_bassiana
XP_001398045.2_Aspergillus_niger
OWW35832.1_Aspergillus_niger
CCU79215.1_argonaute_2_Blumeria_graminis
CCU75107.1_Blumeria_graminis
ESZ94685.1_Sclerotinia_borealis
XP_001598248.1_Sclerotinia_sclerotiorum
XP_011394903.1_Neurospora_crassa
XP_001905964.1_Podospora_anserina
XP_003714217.1_Pyricularia_oryzae
QDE54827.1_argonaute2_Verticillium_nonalfalfae
XP_009648711.1_Verticillium_dahliae
XP_008094315.1_Colletotrichum_graminicola
KFH46392.1_Acremonium_chrysogenum
CRV2T00000975_1_Similar_to_ago2
XP_003052582.1_Nectria_haematococca
EGU84615.1_Fusarium_oxysporum
XP_011319975.1_Fusarium_graminearum
EFZ02610.1_Metarhizium_robertsii
EFY90521.1_Metarhizium_acridum
EHK44328.1_Trichoderma_atroviride
EGR47217.1_Trichoderma_reesei
EHK23898.1_Trichoderma_virens
EJP67294.1_Beauveria_bassiana
XP_003854286.1Zymoseptoria_tritici
XP_003856889.1_Zymoseptoria_tritici
ESZ93307.1_Sclerotinia_borealis
CCF50705.1_Ustilago_hordei
XP_003329577.2_Puccinia_graminis
XP_003326892.2_Puccinia_graminis
NP_587782.1_Schizosaccharomyces_pombe
QDE54826.1_argonaute_1_Verticillium_nonalfalfae
XP_003005611.1_Verticillium_alfalfae
XP_009657937.1_Verticillium_dahliae
XP_008095728.1_Colletotrichum_graminicola
KFH41842.1_Acremonium_chrysogenum
CRV2T00002735_1_Similar_to_ago1
XP_003053663.1_Nectria_haematococca
EGU79915.1_Fusarium_oxysporum
XP_011316005.1_Fusarium_graminearum
EJP69461.1_Beauveria_bassiana
EFY99680.1_Metarhizium_robertsii
EFY86521.1_Metarhizium_acridum
EHK23546.1_Trichoderma_virens
EGR48995.1_Trichoderma_reesei
EHK43969.1_Trichoderma_atroviride
XP_958586.1_Neurospora_crassa
XP_001904709.1_Podospora_anserina
XP_003716704.1_Pyricularia_oryzae
XP_003848385.1_Zymoseptoria_tritici
CCU74167.1_argonaute1_Blumeria_graminis
ESZ91253.1_Sclerotinia_borealis
XP_001586694.1_Sclerotinia_sclerotiorum
XP_001399795.2_Aspergillus_niger
0.4

**Figure S2E**

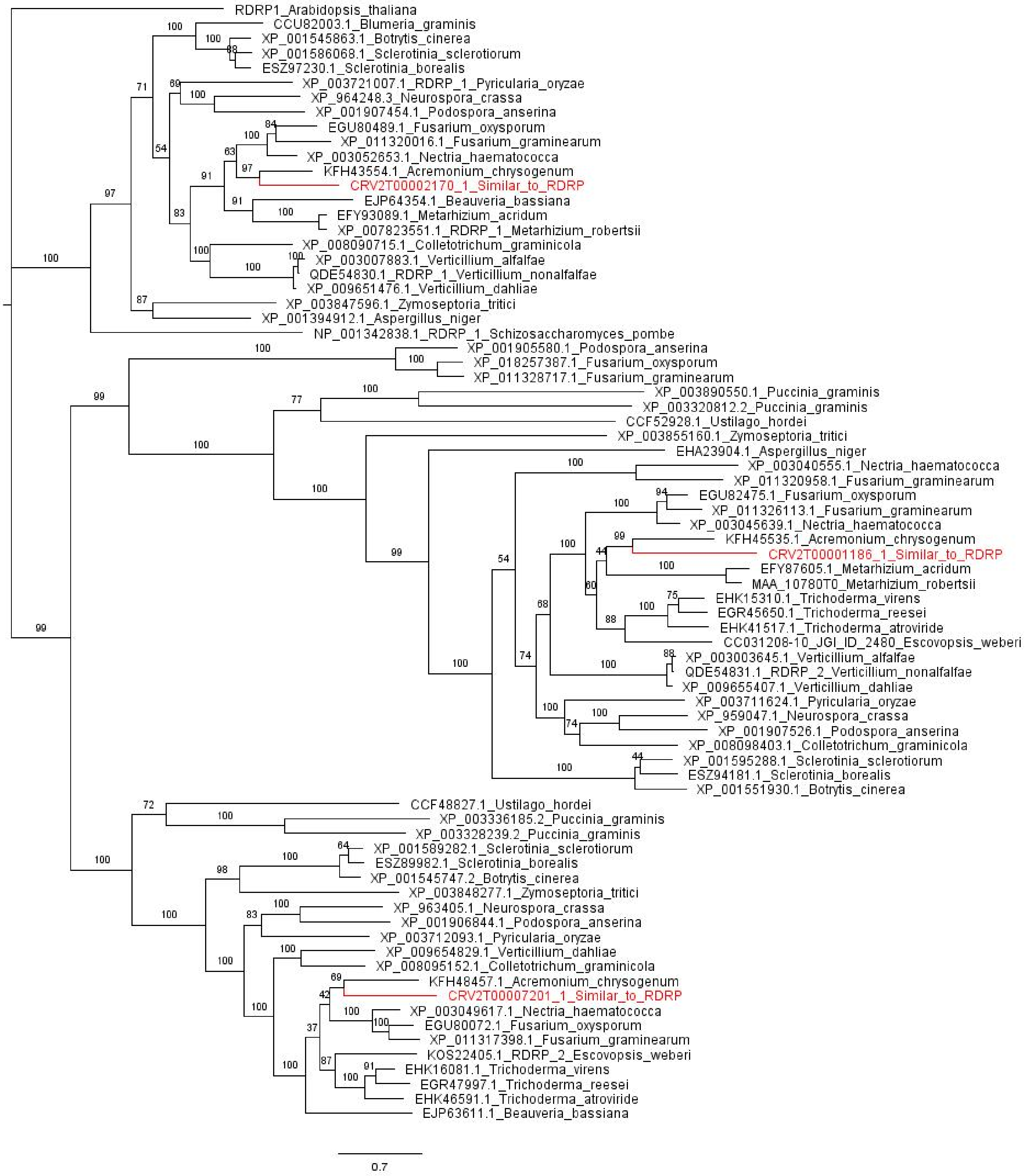


**Figure S2 C-E**: phylogenetic trees presenting the evolutionary relationship among dicer like proteins (C), argonauts (D) and RNA dependent RNA polymerases (E) of several fungal plant pathogens. The trees were generated with iqtree v.1.6.12 and visualized with figtree v.1.4.4.

**Figure S3A**

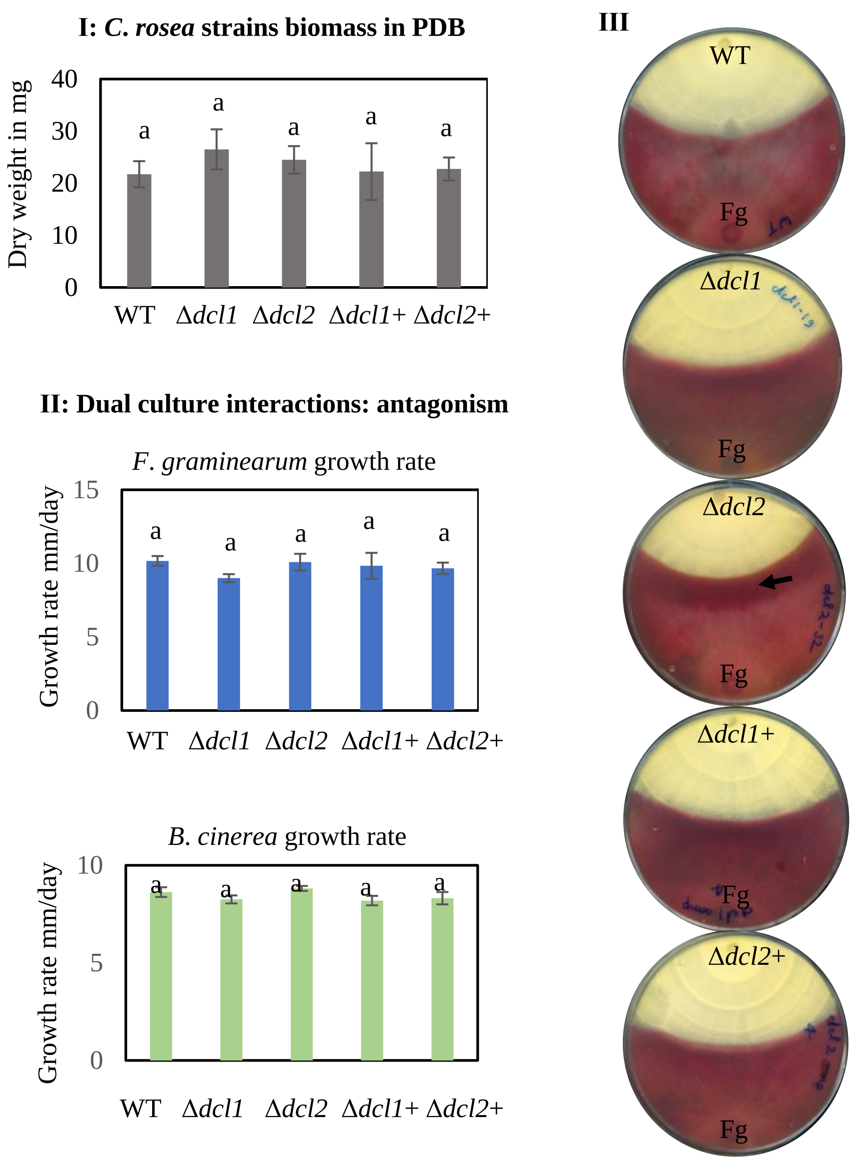


**Figure S3A:** Phenotypic characterization of *C. rosea* strains. (I) Mycelial biomass of *C. rosea* strains in PDB medium. (II) Dual culture assay to analyze antagonistic interactions against *B . cinerea* and *F. graminearum*. Agar plugs of *C . rosea* strains were inoculated on opposite sides in nine cm diameter agar plates and incubated at 25°C. After seven days of incubation, a plug of *B. cinerea* or *F . graminearum* was placed at equal distance to the opposite edge of the plate. Growth rate of *B. cinerea* or *F. graminearum* was recorded daily till the mycelial contact. Error bars represent standard deviation based on 4 biological replicates. The experiments were carried out in four biological replicates. Same letters indicate no statistically significant differences ($P \leq 0.05$) within experiments based on the Tukey HSD test. (III) Pigmentation of *F. graminearum* mycelia was altered (indicated by the arrow) during the interactions with Δ*dcl1* strain. The experiment was performed in four biological replicates and representative photographs of back side of the confrontation plates are shown.

## Figure S3B

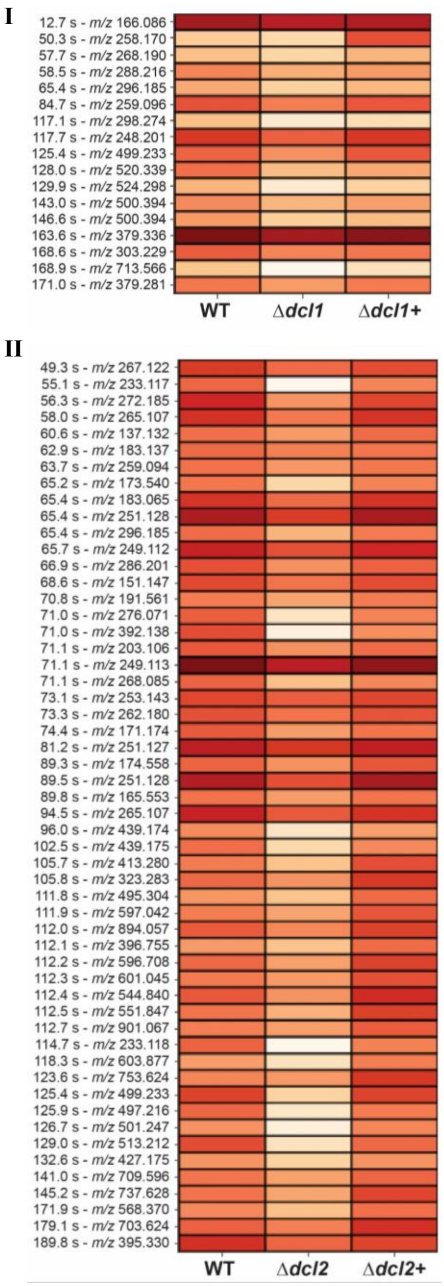


**Figure S3B:** Heatmaps showing all compounds significantly underproduced in the Δ*dcl1*/Δ*dcl2* strains compared to WT and significantly overproduced in the Δ*dcl1+*/Δ*dcl2+* strains compared to the Δ*dcl1*/Δ*dcl2* strains. (**I**) WT/Δ*dcl1*/Δ*dcl1+*. (**II**) WT/Δ*dcl2*/Δ*dcl2+*. Dark red: high concentration. White: low concentration.

**Figure S3C**

**I**

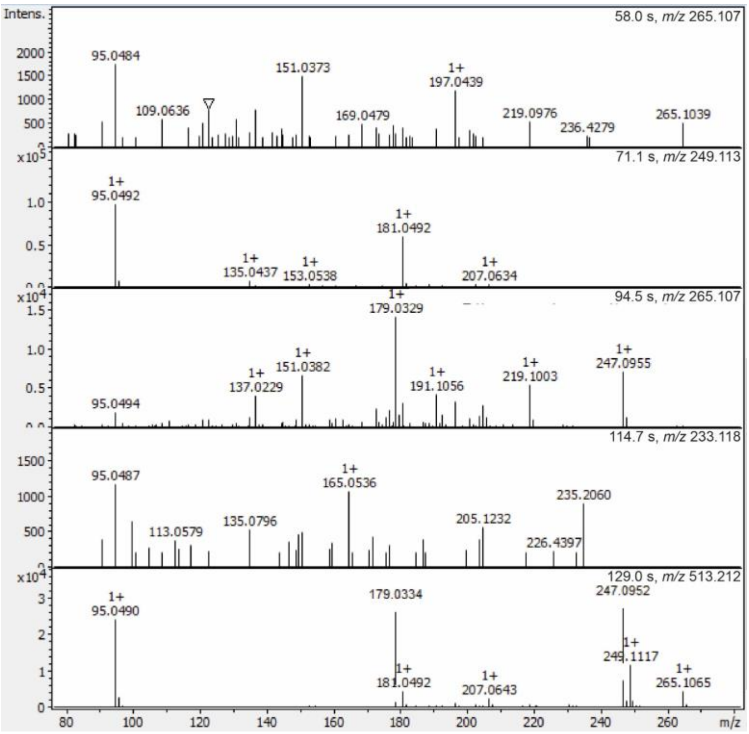


**II**

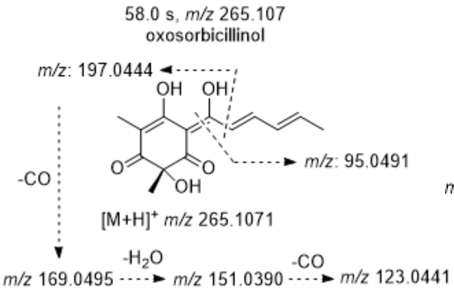


**III**

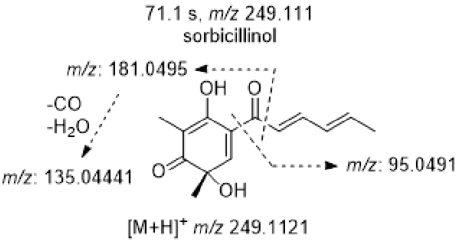

IV

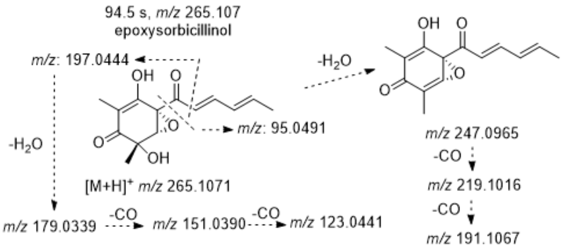


V

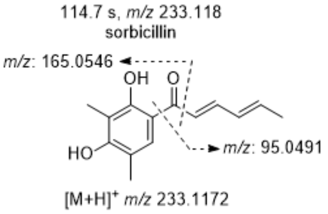


VI

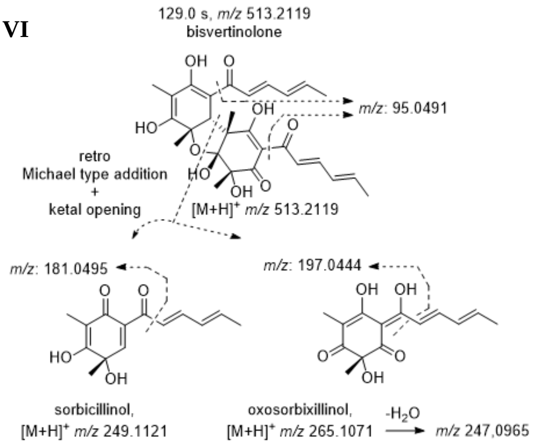


**Figure S3C:** Tentative identification of selected sorbicillin type compounds by UHPLC-MSMS. **(I)** Selected MSMS spectra. **(II-VI)** Proposed formation of observed fragment ions for oxosorbicillinol, sorbicillinol, epoxysorbicillinol, sorbicillin and bisvertinolone, respectively.
